# Schizophrenia: A Narrative Review of Etiopathogenetic, Diagnostic and Treatment Aspects

**DOI:** 10.3390/jcm11175040

**Published:** 2022-08-27

**Authors:** Laura Orsolini, Simone Pompili, Umberto Volpe

**Affiliations:** Unit of Clinical Psychiatry, Department of Clinical Neurosciences/Department of Experimental and Clinical Medicine (DIMSC), Polytechnic University of Marche, 60126 Ancona, Italy

**Keywords:** construct, schizophrenia, schizophrenia spectrum, renaming, rethinking, revising

## Abstract

Although schizophrenia is currently conceptualized as being characterized as a syndrome that includes a collection of signs and symptoms, there is strong evidence of heterogeneous and complex underpinned etiological, etiopathogenetic, and psychopathological mechanisms, which are still under investigation. Therefore, the present viewpoint review is aimed at providing some insights into the recently investigated schizophrenia research fields in order to discuss the potential future research directions in schizophrenia research. The traditional schizophrenia construct and diagnosis were progressively revised and revisited, based on the recently emerging neurobiological, genetic, and epidemiological research. Moreover, innovative diagnostic and therapeutic approaches are pointed to build a new construct, allowing the development of better clinical and treatment outcomes and characterization for schizophrenic individuals, considering a more patient-centered, personalized, and tailored-based dimensional approach. Further translational studies are needed in order to integrate neurobiological, genetic, and environmental studies into clinical practice and to help clinicians and researchers to understand how to redesign a new schizophrenia construct.

## 1. Introduction

Schizophrenia is a severe mental illness (SMI) affecting more than 21 million people worldwide that frequently leads to a persistent disability and impaired cognitive, social, and emotional functioning [1]. Schizophrenia is currently conceptualized as being characterized by at least positive symptoms (such as delusions and hallucinations), negative symptoms (including anhedonia, alogia, avolition, and social withdrawal), and cognitive symptoms (such as deficits in attention, processing speed, verbal learning, visuospatial learning, problem solving, working memory, and cognitive flexibility) [2,3,4,5]. Moreover, social cognition (including emotional intelligence, facial emotion recognition, emotion evaluation, and social inference) impairment may significantly impact the functional recovery in schizophrenia patients, due to the negative effects on interpersonal relationships, community adjustment, and vocational functioning [6,7]. Schizophrenia patients may also experience higher rates of co-occurring medical and/or mental illnesses, such as substance use disorders (mainly alcohol and cannabis), with prevalence rates up to 41% [8]. Due to a disordered lifestyle, an unhealthy diet, a lack of exercise, smoking, the adverse effects of antipsychotic treatment, a limited access to medical care, and the psychiatric illness itself [9,10,11], patients with schizophrenia are more likely to have a metabolic syndrome, a cardiovascular disease, diabetes, other endocrinopathies, an immune disease, and pulmonary illness, in particular, chronic obstructive pulmonary disease [10,11,12,13,14]. The concomitant comorbidity with other mental disorders determines the higher rates in symptomatology relapse, hospitalizations, suicidality, and family and social issues (such as higher rates of incarceration due to mental disorder relapse, treatment discontinuation, higher impulsivity and violent behaviors, and so forth), as well as a higher risk of negative outcomes in the short-term, including higher mortality rates [15,16]. A very recent meta-analysis showed that all causes of mortality were increased in people with schizophrenia, compared to the control group [17]. The specific causes of mortality included suicide, injury, poisoning, pulmonary diseases, endocrine diseases, respiratory diseases, urogenital diseases, diabetes, cancer, and cardio-cerebrovascular causes [17]. Moreover, it has also been found that treatment with an antipsychotic (AP) drug, in particular with second-generation long-acting injectable antipsychotics (SGA-LAIs), seems to be protective against all causes of mortality [17].

However, schizophrenia is a syndrome including a collection of signs and symptoms with heterogeneous etiology, etiopathogenesis, and psychopathological mechanisms that are potentially implicated, with many research directions and pathways currently under investigation [18,19,20]. Nowadays, there are several emerging neurobiological research directions that are suggested to be implicated in the pathogenesis of schizophrenia that could also be helpful in the clinical characterization of the disease, such as the following: (a) genetic factors (e.g., copy number variants [CNV], de novo nonsense genetic mutation, risk genes, polymorphisms in a gene, single nucleotide polymorphisms [SNPs], and so forth) that are implicated in the disrupted development at various stages of fetal life, which program the brain to manifest pre-psychotic features in the prepubertal or puberal age; (b) the neurodevelopmental model of schizophrenia, which considers several non-genetic factors, including perinatal complications, immigration status, and childhood maltreatment and neglect, which could mediate epigenetic changes, potentially determining structural and functional neurodevelopmental aberrations; (c) pathological alterations in multiple brain regions, including the frontal, temporal, parietal, cingulate, and glia components, as well as an excessive synaptic pruning and/or a disruption of neuroplasticity, and so forth; (d) the hypothesis of immune dysfunction and the neuroinflammatory model; (e) many others research pathways, including the emergence of the transdiagnostic model across multiple psychiatric disorders and the different abnormalities that are in the implicated neurotransmitters, such as the dopaminergic and glutammatergic pathways [21,22,23]. Indeed, there is an increased need for a better clinical characterization of individuals who are affected by schizophrenia, considering a more patient-centered, personalized, and tailored-based dimensional approach, which could consider all of the above-mentioned heterogeneous clinical manifestations and endophenotypes of the disease, including the investigation of all of the underpinned genetic and environmental factors [24,25]. Accordingly, the management of schizophrenic individuals should require better data integration towards the personalization of diagnosis and treatment [24,26,27]. Within this context, there have also been recently developed artificial intelligence (AI)- and machine learning (ML)-based approaches, which promise an interesting implementation of statistical tools to build more accurate and precise predictive models of schizophrenia onset, illness course, and potential therapeutic outcomes [28]. These can also identify candidate variables that are putative to be characteristics of schizophrenia spectrum disorders, by allowing a personalized diagnosis, such as a set of resting-state electroencephalographic (EEG) quantitative features, and magnetic resonance imaging of structural and functional anomalies, and so forth [29,30,31].

Therefore, due to the growing knowledge in schizophrenia research and the underpinned mechanisms, we aimed to provide some insights into and a viewpoint on the recently investigated schizophrenia research fields in order to discuss the potential future research directions in schizophrenia research, including the overview of recently developed new constructs and implemented classificatory systems.

## 2. Definitions and Concepts on Schizophrenia

While the cluster of symptoms that clinically define the schizophrenia concept has been noted historically before the 1990s, schizophrenia scientific research was mainly developed following the studies that were carried out by the German psychiatrist Emil Kraeplin (1856–1926) who identified a set of symptoms related to the schizophrenia disease in his *Psychiatrie* manual, which provided a descriptive classification of mental disorders that were based on his clinical observations and experience [32]. In his essay, he identified a set of mental disorders, which he named ‘*processes of psychic degeneration*’, that were characterized by a rapid development of a mental deterioration (later named ‘*dementia praecox*’) [33]. ‘Dementia praecox’ included catatonic syndrome (characterized by a tensive voluntary motor activity), the hebephrenic syndrome (characterized by a distinctive deteriorative course, based on the importance of silliness and minimal positive psychotic symptoms), and the paranoid dementia (characterized by the presence of hallucinations and delusions). Kraeplin [34] mainly focused on the illness course and the chronicity of the disease, rather than on a set of diagnostic criteria, in describing the concept of the ‘dementia praecox’. Kraeplin [34] defined those individuals as distinct from the insanity of tertiary syphilis or the cyclic, non-deteriorating psychosis of a manic-depressive illness. Accordingly, the dementia praecox diagnosis still contained the illness prognosis [33].

Indeed, Kraepelin’s system of mental diseases substantially contributed to the foundation of the modern psychiatric diagnosis in the Diagnostic and Statistical System of Mental Disorders (DSM) and the International Classification of Diseases (ICD). However, since the schizophrenia construct that was developed by Emil Kraeplin [33], several schizophrenia definitions and concepts have changed considerably over the past century, with an increasing disagreement about the core features of schizophrenia [35,36]. In fact, the originally developed Kraeplenian concept was subsequently revised by the Swiss psychiatrist Eugen Bleuler, who mainly focused, during his lecture at a meeting of the German Psychiatric Association in Berlin on 24 April 1908, on the dissociative symptomatology that is related to the illness [37]. At that meeting, Bleuler indeed argued that dementia praecox was associated with neither dementia nor precociousness and emphasized that the splitting of psychic functioning represented the essential schizophrenia feature [38]. Accordingly, Bleuler mainly described schizophrenia originally as a disorder in which “*emotionally charged ideas or drives attain a certain degree of autonomy so that the personality falls into pieces. These fragments can then exist side by side and alternately dominate the main part of the personality, the conscious part of the patient*” [37]. Accordingly, he coined the term “*schizophrenia*” (which was derived from the Greek verb ‘schizein’, indicating splitting, and ‘phren’ denoting the ‘soul, spirit, mind’). Bleuler also stated that schizophrenia was primarily represented by a thought and feeling disorder, comprising the ‘4 As’ (alogia, autistic isolation, ambivalence, and affect blunting) [37,38].

Indeed, the Bleulerian concept of schizophrenia, with the heterogeneity of prognosis and outcomes, indirectly paved the way for later subdivisions of the schizophrenia concept [39]. Consequently, the German psychiatrist Kurt Schneider (1887–1967) proposed a set of fundamental symptoms, named Schneider’s first-rank symptoms (FRS), of which the presence in the subject could be strongly suggestive of a schizophrenia diagnosis [40]. The FRS include the following: (a) auditory hallucinations; (b) thought withdrawal, insertion, and interruption; (c) thought broadcasting; (d) somatic hallucinations; (e) delusional perception; (f) feelings or actions that are made or are influenced by external agents [40]. The FRS are the so-called positive symptoms (i.e., the symptoms that are not usually experienced by people without schizophrenia), and they are usually given priority over other symptoms. In addition, second-rank symptoms include other perceptual disorders, delusional intuition, mood changes, affective flattening, perplexity, and other negative symptoms that represent the deficits of emotional responses and other thought processes [40]. The Schneiderian FRS, which were initially retained in the fourth edition of the Diagnostic and Statistical Manual of Mental Disorders (DSM) [41] and were included in a special schizophrenia diagnostic status in the 10th edition of the International Classification of Diseases (ICD-10) [42], were later dropped in the DSM-5 [43], the DSM-5-TR [44], and in the ICD-11 [45].

The “Neo-Kraepelinian” movement of the 1960s and 1970s argued for the empirical psychometric validation of psychiatric syndromes and posed the basis for the proposal of schizophrenia diagnostic criteria, which was subsequently integrated into both the DSM and ICD versions. Within this context, John Feighner and his colleagues, Eli Robins, Samuel Guze, and George Winokur, at Washington University in St. Louis, Missouri, proposed the Feighner criteria, i.e., a set of influential psychiatric diagnostic criteria that was also developed for schizophrenia diagnosis [46]. In particular, Feighner et al. [46] required as essential criteria for a schizophrenia diagnosis the persistence of a limited set of symptoms (i.e., delusions, hallucinations, or thought disorders) for at least six months, without the return to the premorbid level of psychosocial adjustment. The Feighner criteria were later further expanded with the development of a set of specific diagnostic criteria (namely, research diagnostic criteria (RDC)) [46], which constituted the basis for the DSM-III, as developed by the American Psychiatric Association [47]. The RDC were, indeed, widely used in order to study a variety of schizophrenia-related research issues, particularly those that were related to genetics, psychobiology, and treatment outcomes [48].

Crow [49] simplified the schizophrenia description in terms of a positive form (type I schizophrenia syndrome) and a negative form (type II schizophrenia syndrome, occurring in the absence of positive symptoms), despite the fact that many patients with the type I syndrome can later acquire the features of the type II syndrome, and some patients can have both from an early stage. Type II syndrome is usually associated with the worst prognosis, corresponding more closely to the classical Kraepelinian schizophrenia diagnosis [49]. In addition, Carpenter et al. [50] distinguished between primary and secondary negative symptoms by reviving the long-standing question concerning the primary core deficits. More recently, Andreasen [51] more deeply investigated the negative symptoms that were originally described by Kraepelin [34] and Bleuler [37] as schizophrenic core symptoms. Both Andreasen [51] and Carpenter et al. [50] further investigated the originally developed Bleulerian concept of “thought disorder” as the primary defining feature of schizophrenia, rather than the presence of signs and symptoms such as delusions and hallucinations. Accordingly, Andreasen [51] proposed a neo-Bleulerian unitary model for schizophrenia, defining it as a neurodevelopmentally derived “misconnection syndrome” involving connections between the cortical regions and the cerebellum that are mediated through the thalamus (the cortico–cerebellar–thalamic–cortical circuit).

Meehl [52] proposed a model of the causes and the pathogenesis of schizophrenia and its related states, which emphasized on the presence of a genetically determined aberration in neural transmission that could be potentially responsible of the emergence of schizophrenia and non-psychotic schizotypal states within the diathesis-stressor framework [53]. Gottesman et al. [54] introduced the concept of the ‘epigenetic puzzle’ in schizophrenia, by proposing an explanatory model comprising the different causes of schizophrenia for etiological and phenomenological heterogeneity in schizophrenia [55]. Crow [56] proposed the viral hypothesis of schizophrenia, as derived by a mutagenesis that is caused by viral integration or transposition in human genomic DNA. Following studies that were carried out on subgroups of the non-psychotic relatives of patients who were affected with schizophrenia who displayed defects or abnormalities in clinical, cognitive, biological, social, and other dimensions of functioning that were similar to those shown in schizophrenic individuals [57,58], the hypothesis of schizophrenia liability syndrome [59] was proposed. In fact, based on Paul Meehl’s conceptualization of ‘schizotaxia’ [52], Stone et al. [59] reformulated the concept of liability syndrome based on observable, clinically meaningful symptoms involving the negative symptoms and neurocognitive deficits in non-psychotic relatives [60]. Furthermore, from a more phenomenological perspective, it has been hypothesized that, in schizophrenia spectrum disorders, a profound transformation of subjectivity antedating the onset of major symptoms is accompanied by micro-experiences of self-alienation (e.g., derealization, perplexity, depersonalization, reduced self-presence, and an alteration of the stream of thought) [61]. The self-experiences, indeed, represent fundamental and enduring (more a trait-like feature) distortions of subjectivity, which typically emerge in late childhood and early adolescence [61].

Finally, recent evidence supports the concept that schizophrenia represents a multifactorial disorder that results from a complex interplay between additive and interactive genetic and environmental determinants [62], displaying a highly variable and heterogeneous clinical presentation [63]. Therefore, due to the absence of clear boundaries and the multiplicity of implicated etiological factors, pathophysiological mechanisms, and hypotheses [64,65,66], the schizophrenia concept has been more recently broadened to a spectrum concept in the DSM-5 (and the recently released DSM-5-TR) [43,44] or as a primary psychosis in the ICD-11 [39,45].

## 3. The Heterogeneity and the New Nosological Schizophrenia Constructs

The heterogeneity of schizophrenia resides in the high variability of the phenotypic and clinical expression, with highly varying degrees of functionality, symptoms and personal recovery, and outcomes across individuals, together with a variable range of underlying neurobiological abnormalities, which are potentially implicated in its pathogenesis [67,68]. Indeed, the multifactorial nature of the etiological factors has worsened the difficulty in addressing the causal mechanisms in the disease pathophysiology of the illness [69,70]. However, Tandon et al. [36] exhorted that “*heterogeneity cannot just be an explanation for our failure, but is a problem to be explained*”. Indeed, Carpenter [71] first proposed that the schizophrenia construct should be reconstructed according to the following four major targets: (a) the identification of patient subgroups in order to enhance homogeneity; (b) deconstructing the traditional schizophrenia construct by identifying the specific core psychopathology domains; (c) deconstructing schizophrenia at the levels of neural circuits and behavioral constructs; (d) considering the different stages from the vulnerability of development to the illness onset and disease progression.

Indeed, the traditional schizophrenia construct has elicited a continual debate as the concept has fluctuated across the years, according to the different psychopathological perspectives and the emerging advances in multiple areas of schizophrenia research (e.g., genomics, neuroimaging, epidemiology, and cognitive science) [72]. One of the major obstacles of the traditional schizophrenia construct regards the fact that disorders continue to be defined almost exclusively by a set of symptoms and signs, despite the association between the specific diagnostic categories and biological or behavioral measures having been proven to be modest or inconsistent, therefore, not allowing a better understanding of schizophrenia or the development of more effective interventions for the illness [73]. In particular, the inconclusive findings coming from the neurobiological studies have demonstrated the inadequacy of the current schizophrenia diagnosis by underlining how the current nosological construct does not appear to be exhaustive in identifying all of the multiple and potentially different pathophysiological substrates that are implicated within schizophrenia spectrum disorder [74]. However, many experts in schizophrenia research have pointed to continuing to use the traditional schizophrenia construct because of its utility (at least clinically) and the absence of any current better alternative [20,36,39,66,74,75]. Carpenter [75] suggested replacing it with a broader construct of “primary psychosis”, while Gur [63] suggested replacing it with the “psychosis spectrum disorder” construct. On the other hand, Murray and Quattrone [76], Van Os and Goluksuz [77], and Zick et al. [68] proposed to completely eliminate it. The alternative proposed schizophrenia constructs include dimensional-based schizophrenia constructs [78], the hierarchical psychopathological model by Kotov et al. [79], and the biotype architecture [67,68,80,81], which is illustrated below. Therefore, in order to address these issues, an overview of the different diagnostic classificatory systems, from the traditional DSM/ICD to the recently developed alternative/integrative models, has been provided below.

## 4. The Systems of Diagnostic Classification

Overall, the diagnostic classifications have been ad hoc designed in order to address the following purposes: (a) facilitating research into the causes and the treatment of the illnesses; (b) guiding clinical decision making; (c) helping clinicians in more shared communication [82]. However, the extremely variable and discontinuing phenotypic presentation, diagnostic characteristics, illness trajectory, and treatment response in schizophrenic individuals, together with the highest rates of comorbid disorders, limit the feasibility and applicability of the current diagnostic systems and classifications in the clinical decision making practice in regard to schizophrenia [82]. Therefore, although the latest versions of the DSM-5-TR [44] and ICD-11 [45] might effectively represent some apparently useful approaches facilitating the information exchange among clinicians, they definitely fail to properly capture the biological and pathophysiological nature of schizophrenic individuals, as well as their phenotypical and clinical heterogeneity; indeed, not allowing for a personalized diagnosis or treatment [36,82]. For instance, neurocognitive deficits, which are commonly a core feature of schizophrenia, are not included in the criterion-based definition in the ICD-11 [45] nor in the DSM-5-TR [36,44]. Furthermore, the ICD-11 [45] also differs from the DSM-5 [43] (and the current DSM-5-TR) [44] according to the minimum duration of symptomatology. The ICD-11 [45] requires a minimum duration period of one month or more, whereas, the DSM-5 (and current DSM-5-TR) [36,44] requires the presence of continuous signs of the disturbances that should persist for at least six months beyond the required additional five months of symptoms, which could include prodromal or residual symptoms [83]. Obviously, the shorter duration requirement that is suggested in the ICD-11 was intended to encourage an earlier treatment in order to improve the patient’s outcome. Both the DSM-5-TR and the ICD-11 require at least two types of schizophrenia symptoms lasting at least one month, even though the ICD-11 also includes the presence of experiences of influence, passivity, or control as a separate core symptom in schizophrenia, which represent disturbances in the ‘ego-world boundary’, including passivity experiences, thought withdrawal, and thought broadcasting [83], which were previously included among Schneider’s FRS [40]. Finally, social processing dysfunction is represented as an integral part of the schizophrenia diagnostic criteria only in the DSM-5 [43] and the current DSM-5-TR [44], but not in the ICD-11 [24,45]. Indeed, although both the DSM-5-TR and ICD-11 incorporate, to a greater or lesser extent, the traditional clinical features that were investigated by Kraepelin [34], Bleuler [37] and Schneider [40], the latest iterations of the DSM and the ICD provide clinicians with dimensional assessments based on the key symptom domains covering the positive, negative, affective, and cognitive symptoms of the schizophrenia. However, as one of the most dominant etiological models for schizophrenia postulated that the illness can represent the final state following abnormal neurodevelopmental processes, which may have started years before the illness onset [84], and that it is possible to identify a schizophrenia spectrum disorder, rather than only the presence or absence of the illness, the current diagnostic systems have a series of limitations [85]. In fact, while the original aim of the current diagnostic systems was to allow clinicians to have a shared and homogeneous information exchange, as well as for research purposes, the traditional diagnostic systems, which are mainly based on a set of symptoms and signs, are not able to incorporate any etiology-based components, neurodevelopmental markers, the genetic liability, the subthreshold schizophrenia vulnerability status (i.e., schizotaxia), or many other currently investigated aspects of the disease [75].

Therefore, beyond these classical/traditional diagnostic systems, the National Institute of Mental Health (NIMH) Research Domain Criteria (RDoC) initiative was first developed in 2009 with the aim to build a new classification system for a better understanding of underlying dimensional processes and the development of psychopathology, by using a dimensional approach [63,82]. The RDoC may effectively provide a bridge between the basic behavioral neuroscience research and clinical research by using a dimensional approach in which each function is quantitatively mapped onto specific brain circuits [63]. The RDoC was conceived as an experimental framework in order to support translational research in psychopathology organized around basic functional domains (e.g., cognition, motivation, and motor activity) [72,86]. The focus of the RDoC program is on the fundamental operations of adaptive behavioral/cognitive and brain functioning (e.g., working memory, fear, and behavior) and psychopathology, according to a perspective in terms of the dysregulation of these systems rather than starting with clinical syndromes and then trying to determine their source/causes. The RDoC investigates the entire dimensions of functioning (i.e., negative valence, positive valence, cognition, social processes, arousal/regulatory systems, and sensorimotor systems) from the normal range to increasingly abnormal extents, and no specific cut points for each disorder are specified in order to facilitate studies on the transitions from normality to the different degrees of pathology [72,86,87].

In addition, other research directions have been proposed to reconceptualize schizophrenia psychopathology as consisting of continuous dimensions of maladaptive behaviors, emotions, and cognitions, with some hierarchical taxonomies of phenotypic psychopathological dimensions proposed [88]. Within this context, the Hierarchical Taxonomy of Psychopathology (HiTOP) consortium aimed to integrate the evidence from studies on the organization of psychopathology in order to overcome the arbitrary boundaries between psychopathology and normality, the diagnostic instability, the frequent co-occurring disorders, the heterogeneity within the same diagnosis, and the lack ability to identify the subthreshold clinical cases [79,89,90,91]. HiTOP was built in order to define psychopathology according to a dimensional approach, which also investigates those individuals with subthreshold symptoms or unusual symptom profiles, with the aim to reduce the heterogeneity within those constructs by grouping related symptoms together, independently, by an established diagnosis [68,79]. According to HiTOP, schizophrenia, schizophreniform disorder, schizoaffective disorder, and schizotypal and paranoid personality disorders reflect elevations on both thought disorders and “detachment” spectra dimensions [89,90,91]. In particular, the “detachment” dimension can be considered as a vulnerability trait for negative symptoms and schizophrenia, also among the relatives of people who are affected with schizophrenia, compared to the relatives of healthy probands or probands with mood disorders [92]. Furthermore, the biotype-based architecture model was investigated with the aim to incorporate the biomarkers for differentiating individual cases by subtype [93]. The Bipolar-Schizophrenia Network for Intermediate Phenotypes (B-SNIP1) consortium sought to identify a broad range of biomarkers encompassing the neurocognitive and physiological correlations, with the aim to distinguish the three leading psychosis diagnoses (i.e., schizophrenia, schizoaffective disorder, and bipolar I disorder with psychosis) [67]. Identifying promising neurobiologically distinct subgroups of psychoses by using biomarkers could support genetic-based etiological investigations and may advance treatment developments [94,95]. Indeed, a biotype-based approach could significantly improve the development of new treatment targets, offering an opportunity to match interventions to pathophysiology and to implement more patient-centered, tailored, and personalized approaches to the disease towards a new precision, as well as personalized psychiatry in schizophrenia research and clinic.

## 5. Schizophrenia and Personalized Psychiatry

The concept of personalized medicine is based upon the hypothesis that each individual is unique, hence, diseases are heterogeneous regarding the specific contributing factors and also the specific treatment outcomes [96]. Personalized psychiatry aims to offer an individual and patient-centered approach, including an individualized clinical characterization (also using the tools of the precision psychiatry, including biomarkers, biotypes, endophenotypes, etc.), as well as tailored and personalized treatments for each individual real patient at the right time [97]. The topic of personalized psychiatry becomes more salient, particularly in the field of schizophrenia research, whereas a more concrete emphasis should be posed to the transdiagnostic conceptualization of psychopathology that is related to primary psychosis and schizophrenia, as already pointed out by Carpenter [98]. Furthermore, the investigation of specific biomarkers would be useful in early diagnosis, in clinical monitoring, and in treatment response [99].

According to the Biomarkers Definitions Working Group [100], a biomarker is “a characteristic that is objectively measured and evaluated as an indicator of normal biological processes, pathogenic processes, or pharmacological responses to a therapeutic intervention”. Biomarkers can be molecular, histologic, derived from brain imaging, or physiologic in nature, being classified as diagnostic, prognostic, and theranostic [101]. Biomarkers are not represented by endophenotypes, which are heritable and are more specifically quantitative traits that are associated with disease liability, that instead explain the relationship between the genotype and the phenotype [102]. Indeed, biomarkers could be used as clinical predictors for schizophrenia, its illness course, and its phase, as well as for its treatment and intervention response [103]. A dimensional-based characterization along the RDoC domain [104,105] can help clinicians to identify the specific biomarkers for schizophrenia risk occurrence throughout the lifespan of the individual [63]. For example, dysregulated immunity and inflammatory processes were reported in schizophrenic individuals, despite the fact that the measurement and repeatability of these biomarkers display several challenges in terms of translating this approach in routine clinical practice [105,106,107,108]. Studies on those targeted on polygenic risk scores suggested better characterizing and identifying a specific subgroup of schizophrenic patients who had ongoing inflammation and immune dysfunction [107,108]. Therefore, blood-based biomarkers, including glucose and triglyceride levels, and pro-inflammatory markers (e.g., interleukin-6, tumor necrosis factor alpha, and so forth) have been investigated; however, their profile seems not to be specific to schizophrenia [79].

Further potential neurophysiological, immune, and endocrine biomarkers have been investigated through proteonomic gene expression (transcriptomic) and neuroimaging studies [63,99,109,110,111], even though these biomarkers have not yet been validated and, for this reason, they continue to be investigated in experimental settings rather than in clinical practice [100,112]. However, despite the fact that several biomarkers, including genetic biomarkers, have been identified or are currently under investigation, they have not yet been effectively implemented into routine clinical practice, mainly due to their inconclusive clinical reliability, with exception of a few pharmacogenetic-guided decision support tools [113]. In this regard, the pharmacogenetic research into antipsychotic drugs has examined a number of genetic variants and only a few polymorphisms have been found to be promising in explaining the therapeutic efficacy and side-effects of antipsychotic drugs in schizophrenia spectrum disorders, such as some polymorphisms in the brain-derived neurotrophic factor (BDNF), in some cytochrome CYP genes, and so forth [114,115]

## 6. Genomics

Genetic epidemiological studies have shown that schizophrenia is highly heritable, despite the fact that it has been better described as an underpinned multifactorial etiology with a complex polygenic genetic architecture [116]. Evidence has supported the role of both common and rare genetic variants that are implicated in the development of schizophrenia, as well as several environmental factors that may contribute to its etiology [63]. Indeed, few causal variants have been clearly identified and most of the genetic associations have not imparted any useful clinical implications [116]. For instance, many genome-wide associated variants (GWAV) have not been identified in genes, possibly indicating that they may have a regulatory role in modifying gene expression or they can represent the expression of quantitative trait loci [117]. Indeed, gene expression can be influenced by both genetic and environmental factors and the differences in gene expression can be a biomarker for the diagnosis, but also for a potential therapeutic target in schizophrenia [118,119,120,121]. However, although recent genome-wide association studies (GWAS) have identified more than 100 genetic risk loci in schizophrenia, they are overall responsible of a small effect on the schizophrenia risk [116]. The polygenic risk score (i.e., the measure of polygenic loading) [122] is able to address the polygenic architecture of schizophrenia and it can quantify the common risk allele burden that is carried by schizophrenia individuals [123,124]. Moreover, it has been suggested that the polygenic risk score may be useful in determining the association between schizophrenia and intermediate phenotypes, such as the brain structural alterations in schizophrenic individuals [125]. However, a recent systematic review by van der Merwe et al. [126] did not find any significant association between the polygenic risk score and the brain structural changes in schizophrenic individuals, suggesting the need for further research directions, particularly in the field of intermediate phenotypes other than altered brain structures. Furthermore, according to a recent systematic review, copy number variant (CNV)-based studies have identified five schizophrenia-associated CNV regions containing genes that were found to be differentially expressed in schizophrenia (i.e., PPP1R2 in 3q29, HSPB1 in 7q11.23, INO80E and YPEL3 in 16p11.2, DHRS11 in 17q12, and SEPT5, RTN4R, and SLC2A11 in 22q11.2) [121]. However, the CNVs in these regions are also associated with neurodevelopmental delays, intellectual disabilities [127,128], and other neuropsychiatric phenotypes, including anxiety (3q29, 7q11.23, and 17q12), autism spectrum disorder (ASD; 3q29, 7q11.23, 16p11.2, 17q12, and 22q), attention-deficit/hyperactivity disorder (ADHD; 7q11.23 and 22q), and bipolar disorder (3q29, 7q11.23, and 17q12), as well as in immune system dysfunction, cardiac pathologies, and many other medical issues [119,120]. The most well-investigated CNV that is associated with an increased risk of schizophrenia is the 22q11.2 deletion syndrome, with it being related to a 25-fold increase in schizophrenia risk [129,130,131,132]. Furthermore, several neurotransmitters (i.e., dopamine, serotonin, and glutamate), acting through metabotropic G protein-coupled receptors (GPCRs), which mediate the intracellular signal transduction and the induction of gene expression in order to exert antipsychotic activity, have been genetically investigated in schizophrenia [133,134]. The genetic studies have identified associations between the SNPs in genes that are related to GPCRs and schizophrenia [135]; in particular, some metabotropic glutamate receptors (mGlu), subtype 3 (mGlu_3_), 5-hydroxytryptamine 2A receptor (5-HTA_2A_), and dopamine D_3_ receptors (DRD_3_). SNPs have been associated with schizophrenia, pathognomic measurable endophenotypes, and the treatment response to specific antipsychotics [136,137,138,139,140]. However, further studies are needed in order to investigate the role of GPCRs SNPs variants in schizophrenia and in the antipsychotic’s treatment response [133,141].

However, beyond the genetic susceptibility, epigenetics (including all postnatal modifications of gene expression that are not associated with changes in DNA sequences, such as DNA methylation, chemical modification of histone proteins, non-coding RNA, and other mechanisms that are involved in epigenetic regulation) have demonstrated that not only genetic factors are implicated in schizophrenia, but more specifically epigenetic factors [142]. Epigenetic factors are derived from the interplay between genetic factors and various environmental factors occurring from the fetal period to the developmental period that may potentially influence and modify the psychopathological trajectory of the illness, as well as other post-developmental factors influencing the onset of schizophrenia through an epigenetic mechanism [143].

## 7. Neuroimaging

A set of specific brain structural abnormalities have been widely reported in schizophrenia spectrum disorders, which are mainly considered to be a brain development disorder [144,145,146]. A large-scale metanalysis has reported a smaller hippocampus volume, together with smaller amygdala, thalamus, nucleus accumbens, and intracranial volumes in patients with schizophrenia compared to controls [144]. Moreover, it has been found that a larger palladium and lateral ventricle volume also occurs, compared to healthy controls [144]. Individuals with schizophrenia have also been reported to have widespread cortical thinning and smaller cortical surface [145]. Cortical thickness reductions are larger in individuals under antipsychotic treatment and are negatively correlated with medication dose, symptoms severity, and duration of illness [145]. Limitations in the imaging studies on schizophrenia are represented by the issue that most of them mainly recruited chronic patients and individuals taking antipsychotic treatment, therefore making it difficult to identify the time of the brain changes and the effect of the treatment exposure [146,147]. Functional neuroimaging studies have shown alterations in the brain metabolism and the blood flow in the frontal, cingulate, parietal, putamen, and sensorimotor regions [148,149,150,151]. Dopamine dysfunction has also been observed in schizophrenic patients. Indeed, dopamine D_2_ receptor density and the occupancy of D_2_ receptors by dopamine has been shown to be increased in schizophrenic patients, along with an increased dopamine transmission [152,153]. For other neurotransmitters, the findings coming from neuroimaging studies are still inconsistent; however, some studies have reported a reduced 5-HT_1_ receptor concentration in the midbrain and pons, reduced 5HT_2_ receptors in the neocortex, and a hypofunction of N-methyl-D-aspartate (NDMA) [154,155]. The data that are currently available on the glutamatergic system are still unclear [156].

Neuroimaging data have been more recently extensively investigated with the aim to identify individuals who are at risk of psychosis at an early stage or a prodromal phase [146,157,158]. High-risk individuals who will subsequently develop psychosis or a schizophrenia spectrum disorder showed several structural and functional brain abnormalities compared to the healthy controls, such as grey matter changes in the frontal, temporal, and cingulate cortices, a reduced integrity of striatal and temporal white matter, subcortical volumes of the thalamus, amygdala, striatum, and cerebellum, and changes in the functional connectivity and network organization [159,160,161,162,163]. Further studies have also investigated, through neuroimaging, whether it is possible to identify some predictors of the response to pharmacological medication [146] by demonstrating that a greater striatal dopamine synthesis, an enlarged gray matter volume, and normal gyrification, as well as an increased brain activity in the fronto-parietal regions may act as potential predictors of a positive response to antipsychotic treatments [164,165,166,167,168].

## 8. Environmental Factors

A set of environmental factors, such as childhood adversity, substance use and misuse, minority and ethnicity status, birth season, urbanity, and pregnancy and/or perinatal complications, have been associated with differential clinical manifestations of schizophrenia spectrum disorders [169,170]. A recent systematic review and meta-analysis assessed the evidence for a gene–environment correlation (genes influencing the likelihood of environmental exposure) between schizophrenia polygenic risk score and childhood adversities, observing only a small effect; however, there are still inconsistent findings that do not allow us to draw definitive conclusions [170]. Meta-analyses have also shown that substance use, particularly continued use, was significantly associated with higher rates of positive psychotic symptoms and a higher likelihood of a history of violence and aggressive behaviors [171,172]. In addition, cannabis use, especially with higher potency cannabis, is associated with an increased risk for schizophrenia [173,174,175,176]. In addition, ethnic minority status is correlated with more severe reality distortion, disorganization, and the onset of negative symptomatology [177].

Moreover, the paradigm of the exposome was only recently investigated in the field of schizophrenia [63,178,179,180]. The exposome represents the entirety of the environmental vulnerability underlying the pathoetiology of schizophrenia spectrum disorders, to which an individual is exposed to throughout their life [178,180,181]. According to the exposome model, environmental factors are bi-directionally interlinked, such that cannabis use is associated with childhood adversity, the effects of urbanicity variables (such as population density, deprivation, etc.) can be modified or influenced by individual level factors, such as cannabis use, exclusion, discrimination, and social adversity [178,180]. Moreover, there is evidence to suggest a dose–response relationship between environmental load scores and the severity of the mental health status, as well as the outcomes [179,180,182,183].

## 9. Schizophrenia Treatment and Interventions

Despite several evidence- and consensus-based schizophrenia guidelines that have been generated over the last decades [184,185,186,187,188], the treatment interventions in schizophrenia research are far from being effective and many factors are involved in treatment response based on theoretical groundings, with some innovative fields of research yet to be implemented [21,185,189]. The current approach to schizophrenia in routine clinical practice worldwide is often stereotyped, being mostly prescribed a second-generation antipsychotic drug [190]. Indeed, antipsychotic treatments for schizophrenic individuals have been demonstrated to be effective in managing the core symptoms of schizophrenia, but also they has been reported to be associated with a decreased risk of all-cause, cardiovascular, and suicide mortality, also, in terms of cumulative antipsychotic exposure, particularly in those patients under clozapine treatment [191,192,193].

Furthermore, from a pharmacological perspective, despite the fact that the dopaminergic system has been hugely investigated in the pathophysiology of schizophrenia and has been guided in initially targeting antipsychotic treatments, there is clinical evidence that dopamine blockade is not effective in managing the negative and cognitive symptoms and, in some schizophrenic patients, it does not improve the positive symptoms either [194,195,196]. Therefore, researchers have recently directed their research interest towards new neurochemical targets, such as the glutamatergic system [194,197,198]. While, on the other hand, from a non-pharmacological perspective, despite the demonstrated evidence-based efficacy of cognitive–behavioral approach [199,200,201,202], its use is still poor in routine clinical practice for schizophrenic individuals [24,203]. In patients with treatment-resistant schizophrenia (TRS), researchers have explored the utility of brain stimulation procedures [204], such as electroconvulsive therapy (ECT), repetitive transcranial magnetic stimulation (rTMS), and deep brain stimulation (DBS). Despite the promising preliminary results [205,206], further studies are needed in order to better understand the potential role of these neuromodulatory techniques in the treatment of TRS patients [204]. Finally, psychosocial interventions and recovery-oriented rehabilitative interventions (e.g., cognitive remediation and metacognitive reflection and insight therapy (MERIT) etc.) have been rapidly developed in order to target cognitive and/or metacognitive deficits that can hamper the functional recovery of schizophrenic patients and in subjects at ultra-high risk of psychosis [203,207,208,209,210], even though they do not seem to be adequately integrated in the mental health services [24,203]. Similarly, family-based interventions and supported employment programs are seldom implemented in routine clinical practice [24,203,211]. In particular, there are also initiatives that are aimed at implementing and favoring social integration, regular employment, and reducing the social exclusion of all individuals who are affected by severe mental illnesses, including schizophrenia, such as the Individual Placement and Support (IPS) initiative [212,213]. The IPS became the standard of supported employment and the only evidence-based employment model for people with schizophrenia, indicating a moderate-to-large effect size [214,215], which has also been confirmed in long-term studies [216]. However, despite the recovery-oriented approach that is needed for the management of schizophrenic patients, a resilience-promoting environment (i.e., an environment that integrates interventions in order to increase a positive outcome, despite adversities, in order to implement wellbeing [217]) is often missing in many mental health services [24].

## 10. Discussion

Overall, schizophrenia could better represent an encompassing term referring to a group of related disorders, which have distinct etiologies and that require different treatment strategies [55]. Schizophrenia, indeed, describes a clinical syndrome, not a disease entity. A syndrome consists of co-emerging specific symptoms of unknown etiology and has no clear boundaries with other entities. Symptom dimensions explain more clinical characteristics than diagnostic categories and they are specifically associated with genetic and environmental risk factors that may operate across diagnostic categories [76,116,117,118,119,121,169,170,178,179]. However, the current conceptualization of schizophrenia only appears to be useful to establish evidence-based guidelines for diagnosis and treatment, and to provide valuable information on psychosocial outcomes [39]. In fact, if schizophrenia continues to be defined almost exclusively by a set of symptoms and signs, despite the modest and/or inconsistent association between the diagnostic categories and the biological and/or behavioral measures, the traditional construct of schizophrenia will not be able to clearly reach a comprehensive understanding of the disorder, its heterogeneous clinical presentation and treatment outcomes, or the development of more effective treatments [72]. In fact, it has been well documented that the real-life functioning of schizophrenic patients does not exclusively depend on their symptoms and/or signs, but is more strictly related to context-related factors rather than illness-related ones [218,219], by demonstrating an ability to be stable in their relationships after a four-year follow-up, as reported in the multicenter study that was carried out by the Italian Network for Research on Psychoses [220].

Therefore, the current concept and traditional constructs of schizophrenia appear to be not exhaustive enough in explaining the heterogeneity and the complexity, as well as the complex interplaying roles of additive factors (both genetic and environmental determinants) in the pathogenesis of schizophrenia spectrum disorders [67,68,72,81]. Moreover, the current and traditional schizophrenia constructs are not able to adequately provide a clinical characterization, nor a dimensional and personalized approach to the understanding of each individual who is affected by schizophrenia [24,25,63]. However, there is still no relatively easily applicable and precise biologically-based diagnostic technique for schizophrenia that has enough specificity and sensitivity to replace the traditional schizophrenia constructs [20,36]. The highest clinical utility for the diagnosis of severe brain diseases, such as schizophrenia, is still provided by another brain, the long-term trained brain of a psychiatrist [221]. However, it has been also proposed that a precise psychiatry-based approach could better clinicians to move from a categorical (i.e., ICD and DSM-based criteria) to a dimensional approach in order to better identify people who are at risk for schizophrenia onset, and better clinically and psychopathologically characterize individuals who are affected with schizophrenia spectrum disorder [63,72,86,87,98,99]. However, there is still an intense debate in the scientific community and, despite overcoming the categorical approach that could apparently represent the best way to implement knowledge about schizophrenia, the boundaries of the currently termed schizophrenia could be limited to a neurodevelopmental syndrome that is characterized by disorganization, negative and cognitive symptoms, with a significant presence of anomalous self-experiences that may be distinguishable from other forms of psychosis [222,223].

Our current knowledge and understanding of schizophrenia have been influenced by its multi-level and multi-causal etiology, and the advances in deepening our understanding of its underpinned neurobiology and genetics [63,94,95,99,109,110,111,224]. Therefore, transdiagnostic psychosis spectrum and multi-dimensional frameworks, or multiple functional domains whose combinations comprise significant biotypes that are associated with schizophrenia, have been proposed as replacements for the schizophrenia construct due to the many shared characteristics and the blurring of boundaries between schizophrenia and related entities [36,67,68,78,79,80,94].

Furthermore, advances in multiple areas of neurosciences, including genomics, neuroimaging, cognitive science, and epidemiology, have facilitated the emergence of new conceptions and constructs of schizophrenia, and have allowed us to bridge animal and human research in order to probe the underlying mechanisms of typical and abnormal behaviors in schizophrenia [63]. The genomic data provide increasing support for the concept of systematic transdiagnostic components of neurodevelopmental spectra in schizophrenia [130], although the high heritability has not been translated into satisfying evidence for genetic lesions. In fact, both GWAS- and CNV-based studies that were looking for common genetic variants that are associated with schizophrenia were disappointing, either because the early findings failed to replicate or the large-scale studies failed to detect genome-wide significance [68,94].

Finally, considering that schizophrenia is a severe mental illness that is most strongly associated with stereotyping, prejudice, and a stigmatizing attitude [63], recently several researchers have proposed renaming the word ‘schizophrenia’ (etymologically meaning ‘*split mind*’) [63,68,225,226]. A recent systematic review has demonstrated that renaming schizophrenia could be associated with improvements in attitudes towards patients who are affected with the illness and may increase early diagnosis, mental health access, and reduce stigmatizing behaviors towards the disease and the patients who are affected [227]. Moreover, two recent large surveys of stakeholders demonstrated that approximately 75% of participants agreed to change the name, with the hope of reducing the stigma and the discrimination [228]. Accordingly, some authors have proposed to substitute it with the expression ‘*psychosis spectrum syndrome*’ or ‘*psychosis spectrum illness (PSI*)’, which would be further characterized by key temporal features, such as the age of onset (i.e., childhood, adolescent, or adult), the symptom onset (i.e., acute/insidious), the illness course (i.e., single episode, intermittent, remitting/relapsing, or persistent), and the phase of the illness (i.e., clinical high risk, first episode, recent-onset/early phase, ongoing, or recovered), and so forth [68]. Furthermore, different names have been proposed to refer to schizophrenia in other countries [39]. For instance, the Taiwanese Society of Psychiatry introduced a new name for schizophrenia that means “disorder with dysfunction in thought and perception” in 2012 [229]. In 2022, the Japanese Society of Psychiatry and Neurology renamed the Japanese translation of schizophrenia from “seishin-bunretsu-byo” (meaning mind-split disease) to “togo-shitcho-sho” (meaning integration disorder) [230]. In addition, the Korean Neuropsychiatric Association changed the original Korean name for schizophrenia “jeongshin-bunyeol-byung” (meaning mind-split disorder) to “johyun-byung” (meaning attunement disorder) [231]. Several studies have demonstrated that renaming has significantly modified the attitude toward schizophrenia in health professionals and in the general population [217,231,232]. However, despite these pro-renaming movements, other authors have still declared themselves to be against changing the name for schizophrenia by supporting the idea that changing the name of the condition (or even abolishing the concept) will not affect the root cause of the stigma and will not provide clinicians with a more complete understanding of the causes and the pathophysiological mechanisms underlying schizophrenia [233,234,235].

Therefore, current emerging research supports the need to revise the schizophrenia concept, to implement and readapt the traditional and original schizophrenia constructs by developing new integrative, personalized approaches, to consider the unicity of each individual, the need to clinically characterize the illness onset, the clinical course, the clinical manifestation, the phenotypes, and to personalize the treatment interventions towards a better personalized and dimensional psychiatry. Furthermore, there is also the need to think about renaming, not only the schizophrenia concept, from a neurobiological perspective, but also renaming the term, in order to facilitate a changing mind of health professionals and of the general population.

## Data Availability

Not applicable.

## References

[1] WHO (2018). Key Factors and Publications Concerning Schizophrenia.

[2] McCutcheon R.A., Reis Marques T., Howes O.D. (2020). Schizophrenia—An Overview. JAMA Psychiatry.

[3] Menon V. (2020). Brain Networks and Cognitive Impairment in Psychiatric Disorders. World Psychiatry.

[4] Moritz S., Silverstein S.M., Dietrichkeit M., Gallinat J. (2020). Neurocognitive Deficits in Schizophrenia Are Likely to Be Less Severe and Less Related to the Disorder than Previously Thought. World Psychiatry.

[5] Moura B.M., Isvoranu A.M., Kovacs V., Van Rooijen G., Van Amelsvoort T., Simons C.J.P., Bartels-Velthuis A.A., Bakker P.R., Marcelis M., De Haan L. (2022). The Puzzle of Functional Recovery in Schizophrenia-Spectrum Disorders—Replicating a Network Analysis Study. Schizophr. Bull..

[6] Alston M., Bennett C.F., Rochani H. (2019). Treatment Adherence in Youth with First-Episode Psychosis: Impact of Family Support and Telehealth Delivery. Issues Ment. Health Nurs..

[7] Green M.F., Lee J., Wynn J.K. (2020). Experimental Approaches to Social Disconnection in the General Community: Can We Learn from Schizophrenia Research?. World Psychiatry Off. J. World Psychiatr. Assoc. WPA.

[8] Hunt G.E., Large M.M., Cleary M., Lai H.M.X., Saunders J.B. (2018). Prevalence of Comorbid Substance Use in Schizophrenia Spectrum Disorders in Community and Clinical Settings, 1990–2017: Systematic Review and Meta-Analysis. Drug Alcohol. Depend..

[9] Mamakou V., Thanopoulou A., Gonidakis F., Tentolouris N., Kontaxakis V. (2018). Schizophrenia and Type 2 Diabetes Mellitus. Psychiatr. Psychiatr..

[10] Brink M., Green A., Bojesen A.B., Lamberti J.S., Conwell Y., Andersen K. (2019). Excess Medical Comorbidity and Mortality across the Lifespan in Schizophrenia: A Nationwide Danish Register Study. Schizophr. Res..

[11] Nielsen R.E., Banner J., Jensen S.E. (2021). Cardiovascular Disease in Patients with Severe Mental Illness. Nat. Rev. Cardiol..

[12] Pouget J.G., Han B., Wu Y., Mignot E., Ollila H.M., Barker J., Spain S., Dand N., Trembath R., Martin J. (2019). Schizophrenia Working Group of the Psychiatric Genomics Consortium; Cross-Disorder Analysis of Schizophrenia and 19 Immune-Mediated Diseases Identifies Shared Genetic Risk. Hum. Mol. Genet..

[13] Melkersson K. (2020). Schizophrenia- or Schizoaffective Disorder Diagnosis and the Risk for Subsequent Type 1- or Type 2 Diabetes Mellitus: A Swedish Nationwide Register-Based Cohort Study. Neuro. Endocrinol. Lett..

[14] Misiak B., Stańczykiewicz B., Wiśniewski M., Bartoli F., Carra G., Cavaleri D., Samochowiec J., Jarosz K., Rosińczuk J., Frydecka D. (2021). Thyroid Hormones in Persons with Schizophrenia: A Systematic Review and Meta-Analysis. Prog. Neuropsychopharmacol. Biol. Psychiatry.

[15] Drake R.E., Xie H., McHugo G.J. (2020). A 16-year Follow-up of Patients with Serious Mental Illness and Co-occurring Substance Use Disorder. World Psychiatry.

[16] Plana-Ripoll O., Musliner K.L., Dalsgaard S., Momen N.C., Weye N., Christensen M.K., Agerbo E., Iburg K.M., Laursen T.M., Mortensen P.B. (2020). Nature and Prevalence of Combinations of Mental Disorders and Their Association with Excess Mortality in a Population-Based Cohort Study. World Psychiatry Off. J. World Psychiatr. Assoc. WPA.

[17] Correll C.U., Solmi M., Croatto G., Schneider L.K., Rohani-Montez S.C., Fairley L., Smith N., Bitter I., Gorwood P., Taipale H. (2022). Mortality in People with Schizophrenia: A Systematic Review and Meta-Analysis of Relative Risk and Aggravating or Attenuating Factors. World Psychiatry.

[18] van Os J., Linscott R.J., Myin-Germeys I., Delespaul P., Krabbendam L. (2009). A Systematic Review and Meta-Analysis of the Psychosis Continuum: Evidence for a Psychosis Proneness-Persistence-Impairment Model of Psychotic Disorder. Psychol. Med..

[19] Insel T.R. (2010). Rethinking Schizophrenia. Nature.

[20] Barch D.M., Karcher N., Moran E. (2022). Reinventing Schizophrenia-Embracing Complexity and Complication. Schizophr. Res..

[21] Chekroud A.M., Bondar J., Delgadillo J., Doherty G., Wasil A., Fokkema M., Cohen Z., Belgrave D., DeRubeis R., Iniesta R. (2021). The Promise of Machine Learning in Predicting Treatment Outcomes in Psychiatry. World Psychiatry.

[22] DeLisi L.E. (2022). Redefining Schizophrenia through Genetics: A Commentary on 50 Years Searching for Biological Causes. Schizophr. Res..

[23] Nasrallah H.A. (2022). Re-Inventing the Schizophrenia Syndrome: The Elusive “Theory of Everything”. Schizophr. Res..

[24] Maj M., van Os J., De Hert M., Gaebel W., Galderisi S., Green M.F., Guloksuz S., Harvey P.D., Jones P.B., Malaspina D. (2021). The Clinical Characterization of the Patient with Primary Psychosis Aimed at Personalization of Management. World Psychiatry Off. J. World Psychiatr. Assoc. WPA.

[25] Tortorella A. (2021). We Should Improve Personalization of Management in Patients with a Diagnosis of Schizophrenia. J. Clin. Med..

[26] Owoeye O., Kingston T., Scully P.J., Baldwin P., Browne D., Kinsella A., Russell V., O’Callaghan E., Waddington J.L. (2013). Epidemiological and Clinical Characterization Following a First Psychotic Episode in Major Depressive Disorder: Comparisons with Schizophrenia and Bipolar I Disorder in the Cavan-Monaghan First Episode Psychosis Study (CAMFEPS). Schizophr. Bull..

[27] Tamminga C.A., Ivleva E.I., Keshavan M.S., Pearlson G.D., Clementz B.A., Witte B., Morris D.W., Bishop J., Thaker G.K., Sweeney J.A. (2013). Clinical Phenotypes of Psychosis in the Bipolar-Schizophrenia Network on Intermediate Phenotypes (B-SNIP). Am. J. Psychiatry.

[28] Iniesta R., Stahl D., McGuffin P. (2016). Machine Learning, Statistical Learning and the Future of Biological Research in Psychiatry. Psychol. Med..

[29] Tikka S.K., Singh B.K., Nizamie S.H., Garg S., Mandal S., Thakur K., Singh L.K. (2020). Artificial Intelligence-Based Classification of Schizophrenia: A High Density Electroencephalographic and Support Vector Machine Study. Indian J. Psychiatry.

[30] Lai J.W., Ang C.K.E., Acharya U.R., Cheong K.H. (2021). Schizophrenia: A Survey of Artificial Intelligence Techniques Applied to Detection and Classification. Int. J. Environ. Res. Public. Health.

[31] Cortes-Briones J.A., Tapia-Rivas N.I., D’Souza D.C., Estevez P.A. (2022). Going Deep into Schizophrenia with Artificial Intelligence. Schizophr. Res..

[32] Kraepelin E. (1889). Psychiatrie: Ein kurzes Lehrbuch für Studirende und Aerzte. Dritte, Vielfach Umgearbeitete Auflage.

[33] Kraepelin E. (1893). Psychiatrie: Ein kurzes Lehrbuch für Studirende und Aerzte. Vierte, Vollstandig Umgerbeitete Auflage.

[34] Kraepelin E., Robertson G.M., Barclay R.M. (1919). Dementia Praecox and Paraphrenia. Translated from the 8th German Edition of the Lehrbruch der Psychiatrie.

[35] Heckers S., Kendler K.S. (2020). The Evolution of Kraepelin’s Nosological Principles. World Psychiatry Off. J. World Psychiatr. Assoc. WPA.

[36] Tandon R. (2022). Agreement on the Contours of Schizophrenia: The First Order of Business. Schizophr. Res..

[37] Bleuler E. (1911). Dementia Praecox or the Group of Schizophrenias.

[38] Kuhn R. (2004). Eugen Bleuler’s Concepts of Psychopathology. Hist. Psychiatry.

[39] Gaebel W., Salveridou-Hof E. (2022). Reinventing Schizophrenia: Updating the Construct-Primary Schizophrenia 2021—The Road Ahead. Schizophr. Res..

[40] Schneider K. (1959). Clinical Psychopathology.

[41] APA (2020). Diagnostic and Statistical Manual of Mental Disorders.

[42] WHO (1992). International Statistical Classification of Diseases and Related Health Problems.

[43] APA (2013). Diagnostic and Statistical Manual of Mental Disorders.

[44] APA (2022). Diagnostic and Statistical Manual of Mental Disorders.

[45] WHO (2019). International Statistical Classification of Diseases and Related Health Problems, 11th.

[46] Feighner J.P., Robins E., Guze S.B., Woodruff R.A., Winokur G., Munoz R. (1972). Diagnostic Criteria for Use in Psychiatric Research. Arch. Gen. Psychiatry.

[47] APA (1980). Diagnostic and Statistical Manual of Mental Disorders.

[48] Spitzer R.L., Endicott J., Robins E. (1978). Research Diagnostic Criteria: Rationale and Reliability. Arch. Gen. Psychiatry.

[49] Crow T.J. (1980). Molecular Pathology of Schizophrenia: More than One Disease Process?. Br. Med. J..

[50] Carpenter W.T., Heinrichs D.W., Wagman A.M. (1988). Deficit and Nondeficit Forms of Schizophrenia: The Concept. Am. J. Psychiatry.

[51] Andreasen N.C.A. (1999). Unitary Model of Schizophrenia: Bleuler’s “Fragmented Phrene” as Schizencephaly. Arch. Gen. Psychiatry.

[52] Meehl P.E. (1969). Schizotaxia, Schizotypy, Schizophrenia. Schizophrenia: Seven Approaches.

[53] Lenzenweger M.F. (2006). Schizotaxia, Schizotypy, and Schizophrenia: Paul E. Meehl’s Blueprint for the Experimental Psychopathology and Genetics of Schizophrenia. J. Abnorm. Psychol..

[54] Roberts D.F. (1983). Schizophrenia: The Epigenetic Puzzle. By I. I. Gottesman and J. Shields. Cambridge University Press: Cambridge. 1982. Psychol. Med..

[55] Schizophrenia Genesis: The Origins of Madness—Royal Holloway, University of London. https://librarysearch.royalholloway.ac.uk/primo-explore/fulldisplay/44ROY_ALMA_DS2128877520002671/44ROY_VU2.

[56] Crow T.J.A. (1984). Re-Evaluation of the Viral Hypothesis: Is Psychosis the Result of Retroviral Integration at a Site Close to the Cerebral Dominance Gene?. Br. J. Psychiatry J. Ment. Sci..

[57] Stone W.S., Faraone S.V., Seidman L.J., Olson E.A., Tsuang M.T., Consortium on the Genetics on Schizophrenia (2005). Searching for the Liability to Schizophrenia: Concepts and Methods Underlying Genetic High-Risk Studies of Adolescents. J. Child Adolesc. Psychopharmacol..

[58] Keshavan M.S., Kulkarni S., Bhojraj T., Francis A., Diwadkar V., Montrose D.M., Seidman L.J., Sweeney J. (2010). Premorbid Cognitive Deficits in Young Relatives of Schizophrenia Patients. Front. Hum. Neurosci..

[59] Stone W.S., Hsi X., Giuliano A.J., Tan L., Zhu S., Li L., Seidman L.J., Tsuang M.T. (2012). Are Neurocognitive, Clinical and Social Dysfunctions in Schizotaxia Reversible Pharmacologically?: Results from the Changsha Study. Asian J. Psychiatry.

[60] Tsuang M.T., Stone W.S., Faraone S.V. (2000). Toward Reformulating the Diagnosis of Schizophrenia. Am. J. Psychiatry.

[61] Raballo A., Poletti M., Preti A., Parnas J. (2021). The Self in the Spectrum: A Meta-Analysis of the Evidence Linking Basic Self-Disorders and Schizophrenia. Schizophr. Bull..

[62] van Os J., Kenis G., Rutten B.P.F. (2010). The Environment and Schizophrenia. Nature.

[63] Gur R.E. (2022). Considering Alternatives to the Schizophrenia Construct. Schizophr. Res..

[64] Tandon R., Gaebel W., Barch D.M., Bustillo J., Gur R.E., Heckers S., Malaspina D., Owen M.J., Schultz S. (2013). Definition and Description of Schizophrenia in the DSM-5. Schizophr. Res..

[65] Taghia J., Cai W., Ryali S., Kochalka J., Nicholas J., Chen T., Menon V. (2018). Uncovering Hidden Brain State Dynamics That Regulate Performance and Decision-Making during Cognition. Nat. Commun..

[66] Cannon T.D. (2022). Psychosis, Schizophrenia, and States vs. Traits. Schizophr. Res..

[67] Clementz B.A., Trotti R.L., Pearlson G.D., Keshavan M.S., Gershon E.S., Keedy S.K., Ivleva E.I., McDowell J.E., Tamminga C.A. (2020). Testing Psychosis Phenotypes from Bipolar-Schizophrenia Network for Intermediate Phenotypes for Clinical Application: Biotype Characteristics and Targets. Biol. Psychiatry Cogn. Neurosci. Neuroimag..

[68] Zick J.L., Staglin B., Vinogradov S. (2022). Eliminate Schizophrenia. Schizophr. Res..

[69] Namkung H., Kim S.H., Sawa A. (2017). The Insula: An Underestimated Brain Area in Clinical Neuroscience, Psychiatry, and Neurology. Trends Neurosci..

[70] Sawa A., Yang K., Cascella N.G. (2022). Paradigm Shift on the Concept of Schizophrenia That Matches with Both Academic and Clinical Needs. Schizophr. Res..

[71] Carpenter W.T., Silverstein S.M., Moghaddam B., Wykes T. (2013). How the Diagnosis of Schizophrenia Impeded the Advance of Knowledge (and What to Do About It). Schizophrenia: Evolution and Synthesis.

[72] Cuthbert B.N., Morris S.E. (2021). Evolving Concepts of the Schizophrenia Spectrum: A Research Domain Criteria Perspective. Front. Psychiatry.

[73] Kapur S., Phillips A.G., Insel T.R. (2012). Why Has It Taken so Long for Biological Psychiatry to Develop Clinical Tests and What to Do about It?. Mol. Psychiatry.

[74] Dazzan P. (2022). Is Our Mistake Trying to Identify a “Homogeneous” Schizophrenia Construct?. Schizophr. Res..

[75] Carpenter W.T. (2022). Schizophrenia: A View of Immediate Future. Schizophr. Res..

[76] Murray R.M., Quattrone D. (2022). The Kraepelian Concept of Schizophrenia: Dying but Not yet Dead. Schizophr. Res..

[77] van Os J., Guloksuz S. (2022). Schizophrenia as a Symptom of Psychiatry’s Reluctance to Enter the Moral Era of Medicine. Schizophr. Res..

[78] Kendler K.S., Heckers S. (2022). The Schizophrenia Concept. Schizophr. Res..

[79] Kotov R., Jonas K.G., Carpenter W.T., Dretsch M.N., Eaton N.R., Forbes M.K., Forbush K.T., Hobbs K., Reininghaus U., Slade T. (2020). Validity and Utility of Hierarchical Taxonomy of Psychopathology (HiTOP): I. Psychosis Superspectrum. World Psychiatry.

[80] Tamminga C.A. (2022). Assessing Striatal Dopamine in Schizophrenia. Biol. Psychiatry.

[81] Keshavan M.S., Nasrallah H.A., Tandon R. (2011). Schizophrenia, “Just the Facts” 6. Moving Ahead with the Schizophrenia Concept: From the Elephant to the Mouse. Schizophr. Res..

[82] Gordon J.A., Morris S.E., Avenevoli S.A. (2022). Framework for Integration of Dimensional and Diagnostic Approaches to the Diagnosis of Schizophrenia. Schizophr. Res..

[83] First M.B., Gaebel W., Maj M., Stein D.J., Kogan C.S., Saunders J.B., Poznyak V.B., Gureje O., Lewis-Fernández R., Maercker A. (2021). An Organization- and Category-Level Comparison of Diagnostic Requirements for Mental Disorders in ICD-11 and DSM-5. World Psychiatry Off. J. World Psychiatr. Assoc. WPA.

[84] Rapoport J., Giedd J., Gogtay N. (2012). Neurodevelopmental Model of Schizophrenia: Update 2012. Mol. Psychiatry.

[85] Reichenberg A., Akbarian S. (2022). Towards DSM 10: A Bio-Classification of Developmental Schizophrenia?. Schizophr. Res..

[86] Sanislow C.A. (2020). RDoC at 10: Changing the Discourse for Psychopathology. World Psychiatry.

[87] Ross C.A., Margolis R.L. (2019). Research Domain Criteria: Strengths, Weaknesses, and Potential Alternatives for Future Psychiatric Research. Mol. Neuropsychiatry.

[88] Lahey B.B., Moore T.M., Kaczkurkin A.N., Zald D.H. (2021). Hierarchical Models of Psychopathology: Empirical Support, Implications, and Remaining Issues. World Psychiatry.

[89] Kotov R., Krueger R.F., Watson D., Achenbach T.M., Althoff R.R., Bagby R.M., Brown T.A., Carpenter W.T., Caspi A., Clark L.A. (2017). The Hierarchical Taxonomy of Psychopathology (HiTOP): A Dimensional Alternative to Traditional Nosologies. J. Abnorm. Psychol..

[90] Kotov R., Jonas K.G., Lian W., Docherty A.R., Carpenter W.T. (2022). Reconceptualizing Schizophrenia in the Hierarchical Taxonomy of Psychopathology (HiTOP). Schizophr. Res..

[91] Krueger R.F., Hobbs K.A., Conway C.C., Dick D.M., Dretsch M.N., Eaton N.R., Forbes M.K., Forbush K.T., Keyes K.M., Latzman R.D. (2021). HiTOP Utility Workgroup. Validity and Utility of Hierarchical Taxonomy of Psychopathology (HiTOP): II. Externalizing Superspectrum. World Psychiatry Off. J. World Psychiatr. Assoc. WPA.

[92] Hjorthøj C., Albert N., Nordentoft M. (2018). Association of Substance Use Disorders with Conversion From Schizotypal Disorder to Schizophrenia. JAMA Psychiatry.

[93] Clementz B.A., Parker D.A., Trotti R.L., McDowell J.E., Keedy S.K., Keshavan M.S., Pearlson G.D., Gershon E.S., Ivleva E.I., Huang L.Y. (2022). Psychosis Biotypes: Replication and Validation from the B-SNIP Consortium. Schizophr. Bull..

[94] Clementz B.A., Sweeney J.A., Hamm J.P., Ivleva E.I., Ethridge L.E., Pearlson G.D., Keshavan M.S., Tamminga C.A. (2016). Identification of Distinct Psychosis Biotypes Using Brain-Based Biomarkers. Am. J. Psychiatry.

[95] Hall M.H., Smoller J.W., Cook N.R., Schulze K., Hyoun Lee P., Taylor G., Bramon E., Coleman M.J., Murray R.M., Salisbury D.F. (2012). Patterns of Deficits in Brain Function in Bipolar Disorder and Schizophrenia: A Cluster Analytic Study. Psychiatry Res..

[96] Ginsburg G.S., McCarthy J.J. (2001). Personalized Medicine: Revolutionizing Drug Discovery and Patient Care. Trends Biotechnol..

[97] Roffman J.L. (2011). Biomarkers and Personalized Psychiatry. Harv. Rev. Psychiatry.

[98] Carpenter W.T. (2021). Primary Psychosis: More to Know, Much More to Do. World Psychiatry.

[99] Levchenko A., Nurgaliev T., Kanapin A., Samsonova A., Gainetdinov R.R. (2020). Current Challenges and Possible Future Developments in Personalized Psychiatry with an Emphasis on Psychotic Disorders. Heliyon.

[100] FDA-NIH Biomarker Working Group (2016). BEST (Biomarkers, EndpointS, and Other Tools) Resource.

[101] Weickert C.S., Weickert T.W., Pillai A., Buckley P.F. (2013). Biomarkers in Schizophrenia: A Brief Conceptual Consideration. Dis. Markers.

[102] Gottesman I.I., Gould T.D. (2003). The Endophenotype Concept in Psychiatry: Etymology and Strategic Intentions. Am. J. Psychiatry.

[103] Califf R.M. (2018). Biomarker Definitions and Their Applications. Exp. Biol. Med..

[104] de Andrada Pereira B., Wangsawatwong P., Lehrman J.N., Sawa A.G.U., Lindsey D.P., Yerby S.A., Godzik J., Waguespack A.M., Uribe J., Kelly B.P. (2022). Biomechanics of a Laterally Placed Sacroiliac Joint Fusion Device Supplemental to S2 Alar-Iliac Fixation in a Long-Segment Adult Spinal Deformity Construct: A Cadaveric Study of Stability and Strain Distribution. J. Neurosurg. Spine.

[105] Kelly D.L., Buchanan R.W. (2022). Can the Current Schizophrenia Construct Endure?. Schizophr. Res..

[106] Khandaker G.M., Cousins L., Deakin J., Lennox B.R., Yolken R., Jones P.B. (2015). Inflammation and Immunity in Schizophrenia: Implications for Pathophysiology and Treatment. Lancet Psychiatry.

[107] Chen J., Mize T., Wu J.S., Hong E., Nimgaonkar V., Kendler K.S., Allen D., Oh E., Netski A., Chen X. (2020). Polygenic Risk Scores for Subtyping of Schizophrenia. Schizophr. Res. Treat..

[108] Maes M., Sirivichayakul S., Matsumoto A.K., Maes A., Michelin A.P., de Oliveira Semeão L., de Lima Pedrão J.V., Moreira E.G., Barbosa D.S. (2020). Increased Levels of Plasma Tumor Necrosis Factor-α Mediate Schizophrenia Symptom Dimensions and Neurocognitive Impairments and Are Inversely Associated with Natural IgM Directed to Malondialdehyde and Paraoxonase 1 Activity. Mol. Neurobiol..

[109] Fabbri C., Hosak L., Mössner R., Giegling I., Mandelli L., Bellivier F., Claes S., Collier D.A., Corrales A., Delisi L.E. (2017). Consensus Paper of the WFSBP Task Force on Genetics: Genetics, Epigenetics and Gene Expression Markers of Major Depressive Disorder and Antidepressant Response. World J. Biol. Psychiatry Off. J. World Fed. Soc. Biol. Psychiatry.

[110] Gandal M.J., Haney J.R., Parikshak N.N., Leppa V., Ramaswami G., Hartl C., Schork A.J., Appadurai V., Buil A. (2018). CommonMind Consortium; PsychENCODE Consortium; iPSYCH-BROAD Working Group. Shared Molecular Neuropathology across Major Psychiatric Disorders Parallels Polygenic Overlap. Science.

[111] Schmitt A., Martins-de-Souza D., Akbarian S., Cassoli J.S., Ehrenreich H., Fischer A., Fonteh A., Gattaz W.F., Gawlik M., Gerlach M. (2017). Members of the WFSBP Task Force on Biological Markers. Consensus Paper of the WFSBP Task Force on Biological Markers: Criteria for Biomarkers and Endophenotypes of Schizophrenia, Part III: Molecular Mechanisms. World J. Biol. Psychiatry Off. J. World Fed. Soc. Biol. Psychiatry.

[112] Kalia M., Costa E., Silva J. (2015). Biomarkers of Psychiatric Diseases: Current Status and Future Prospects. Metabolism..

[113] Lydon-Staley D.M., Bassett D.S. (2018). Network Neuroscience: A Framework for Developing Biomarkers in Psychiatry. Curr. Top. Behav. Neurosci..

[114] Yenilmez E.D., Tamam L., Karaytug O., Tuli A. (2018). Characterization CYP1A2, CYP2C9, CYP2C19 and CYP2D6 Polymorphisms Using HRMA in Psychiatry Patients with Schizophrenia and Bipolar Disease for Personalized Medicine. Comb. Chem. High Throughput Screen..

[115] Han M., Deng C. (2020). BDNF as a Pharmacogenetic Target for Antipsychotic Treatment of Schizophrenia. Neurosci. Lett..

[116] Owen M.J., Sawa A., Mortensen P.B. (2016). Schizophrenia. Lancet Lond. Engl..

[117] Schizophrenia Working Group of the Psychiatric Genomics Consortium (2014). Biological Insights from 108 Schizophrenia-Associated Genetic Loci. Nature.

[118] Darby M.M., Yolken R.H., Sabunciyan S. (2016). Consistently Altered Expression of Gene Sets in Postmortem Brains of Individuals with Major Psychiatric Disorders. Transl. Psychiatry.

[119] Charney A.W., Stahl E.A., Green E.K., Chen C.Y., Moran J.L., Chambert K., Belliveau R.A., Forty L., Gordon-Smith K., Lee P.H. (2019). Contribution of Rare Copy Number Variants to Bipolar Disorder Risk Is Limited to Schizoaffective Cases. Biol. Psychiatry.

[120] Hubbard L., Rees E., Morris D.W., Lynham A.J., Richards A.L., Pardiñas A.F., Legge S.E., Harold D., Zammit S. (2021). Rare Copy Number Variants Are Associated with Poorer Cognition in Schizophrenia. Biol. Psychiatry.

[121] Merikangas A.K., Shelly M., Knighton A., Kotler N., Tanenbaum N., Almasy L. (2022). What Genes Are Differentially Expressed in Individuals with Schizophrenia? A Systematic Review. Mol. Psychiatry.

[122] Murray G.K., Lin T., Austin J., McGrath J.J., Hickie I.B., Wray N.R. (2021). Could Polygenic Risk Scores Be Useful in Psychiatry?: A Review. JAMA Psychiatry.

[123] Mistry S., Harrison J.R., Smith D.J., Escott-Price V., Zammit S. (2018). The Use of Polygenic Risk Scores to Identify Phenotypes Associated with Genetic Risk of Bipolar Disorder and Depression: A Systematic Review. J. Affect. Disord..

[124] Ronald A., Pain O. (2018). A Systematic Review of Genome-Wide Research on Psychotic Experiences and Negative Symptom Traits: New Revelations and Implications for Psychiatry. Hum. Mol. Genet..

[125] Kauppi K., Westlye L.T., Tesli M., Bettella F., Brandt C.L., Mattingsdal M., Ueland T., Espeseth T., Agartz I., Melle I. (2015). Polygenic Risk for Schizophrenia Associated with Working Memory-Related Prefrontal Brain Activation in Patients with Schizophrenia and Healthy Controls. Schizophr. Bull..

[126] van der Merwe C., Passchier R., Mufford M., Ramesar R., Dalvie S., Stein D.J. (2019). Polygenic Risk for Schizophrenia and Associated Brain Structural Changes: A Systematic Review. Compr. Psychiatry.

[127] Richards A.L., Pardiñas A.F., Frizzati A., Tansey K.E., Lynham A.J., Holmans P., Legge S.E., Savage J.E., Agartz I., Andreassen O.A. (2020). The Relationship Between Polygenic Risk Scores and Cognition in Schizophrenia. Schizophr. Bull..

[128] Legge S.E., Cardno A.G., Allardyce J., Dennison C., Hubbard L., Pardiñas A.F., Richards A., Rees E., Di Florio A., Escott-Price V. (2021). Associations Between Schizophrenia Polygenic Liability, Symptom Dimensions, and Cognitive Ability in Schizophrenia. JAMA Psychiatry.

[129] Costain G., Lionel A.C., Merico D., Forsythe P., Russell K., Lowther C., Yuen T., Husted J., Stavropoulos D.J., Speevak M. (2013). Pathogenic Rare Copy Number Variants in Community-Based Schizophrenia Suggest a Potential Role for Clinical Microarrays. Hum. Mol. Genet..

[130] Owen M.J., O’Donovan M.C. (2017). Schizophrenia and the Neurodevelopmental Continuum: Evidence from Genomics. World Psychiatry Off. J. World Psychiatr. Assoc. WPA.

[131] Marshall C.R., Howrigan D.P., Merico D., Thiruvahindrapuram B., Wu W., Greer D.S., Antaki D., Shetty A., Holmans P.A. (2017). CNV and Schizophrenia Working Groups of the Psychiatric Genomics Consortium. Contribution of Copy Number Variants to Schizophrenia from a Genome-Wide Study of 41,321 Subjects. Nat. Genet..

[132] Cleynen I., Engchuan W., Hestand M.S., Heung T., Holleman A.M., Johnston H.R., Monfeuga T., McDonald-McGinn D.M., Gur R.E., Morrow B.E. (2021). International 22q11.2DS Brain and Behavior Consortium. Genetic Contributors to Risk of Schizophrenia in the Presence of a 22q11.2 Deletion. Mol. Psychiatry.

[133] Komatsu H., Fukuchi M., Habata Y. (2019). Potential utility of Biased GPCR signaling for treatment of psychiatric disorders. Int. J. Mol. Sci..

[134] Boczek T., Mackiewicz J., Sobolczyk M., Wawrzyniak J., Lisek M., Ferenc B., Guo F., Zylinska L. (2021). The role of G Protein-Coupled Receptors (GPCRs) and calcium signaling in schizophrenia. Focus on GPCRs activated by neurotransmitters and chemokines. Cells.

[135] Morozova A., Zorkina T., Pavlov K., Pavlova O., Storozheva Z., Zubkov E., Zakharova N., Karpenko O., Reznik A., Chekjonin V. (2019). Association of rs4680 COMT, rs6280 DRD3, and rs7322347 5HT2A with clinical features of youth-onset schizophrenia. Front. Psuchiatry.

[136] Maj C., Minelli A., Giacopuzzi E., Sacchetti E., Gennarelli M. (2016). The role of metabotropic glutamate receptor genes in schizophrenia. Curr. Neurophamacol..

[137] Saini S.M., Mancuso S.G., Mostaid M.S., Liu C., Pantelis C., Everall I.P., Bousman C.A. (2017). Meta-analysis support GWAS-implicated link between GRM3 and schizophrenia risk. Transl. Psychiatry.

[138] Kang Y., Zhang W., Lv Y., Cai S., Xu H., Wang J., Huang L. (2020). Effects of the 5-HT2A and DRD3 genotypes on cortical morphology and functional connectivity density in drug-naïve first episode schizophrenia. Schizophr. Res..

[139] Zakharyan R., Ghazaryan H., Kocourkova L., Chavushyan A., Mkrtchyan A., Zizkova V., Arakelyan A., Martin P. (2020). Association of genetic variations of dopamine and serotonin in schizophrenia. Arch. Med. Res..

[140] Zhao M., Ma J., Li M., Zhu W., Zhou W., Shen L., Wu H., Zhang N., Wu S., Fu C. (2022). Different responses to risperidone treatment in schizophrenia: A multicenter genome-wide association and whole exome sequencing joint study. Transl. Psychiatry.

[141] Rahman M.M., Mim S.A., Islam M.R., Sultana N., Ahmed M., Kamal M.A. (2022). Role of G-proteins and GPCR-mediated signaling in neuropathophysiology. CSN. Neurol. Disord. Drug. Targets.

[142] Smigielski L., Jagannath V., Rössler W., Walitza S., Grünblatt E. (2020). Epigenetic Mechanisms in Schizophrenia and Other Psychotic Disorders: A Systematic Review of Empirical Human Findings. Mol. Psychiatry.

[143] Radua J., Ramella-Cravaro V., Ioannidis J.P.A., Reichenberg A., Phiphopthatsanee N., Amir T., Yenn Thoo H., Oliver D., Davies C., Morgan C. (2018). What Causes Psychosis? An Umbrella Review of Risk and Protective Factors. World Psychiatry.

[144] van Erp T.G.M., Hibar D.P., Rasmussen J.M., Glahn D.C., Pearlson G.D., Andreassen O.A., Agartz I., Westlye L.T., Haukvik U.K., Dale A.M. (2016). Subcortical Brain Volume Abnormalities in 2028 Individuals with Schizophrenia and 2540 Healthy Controls via the ENIGMA Consortium. Mol. Psychiatry.

[145] van Erp T.G.M., Walton E., Hibar D.P., Schmaal L., Jiang W., Glahn D.C., Pearlson G.D., Yao N., Fukunaga M., Hashimoto R. (2018). Cortical Brain Abnormalities in 4474 Individuals with Schizophrenia and 5098 Control Subjects via the Enhancing Neuro Imaging Genetics Through Meta Analysis (ENIGMA) Consortium. Biol. Psychiatry.

[146] Keshavan M.S., Collin G., Guimond S., Kelly S., Prasad K.M., Lizano P. (2020). Neuroimaging in Schizophrenia. Neuroimaging Clin. N. Am..

[147] Wheeler A.L., Voineskos A.N. (2014). A Review of Structural Neuroimaging in Schizophrenia: From Connectivity to Connectomics. Front. Hum. Neurosci..

[148] Hill K., Mann L., Laws K.R., Stephenson C.M.E., Nimmo-Smith I., McKenna P.J. (2004). Hypofrontality in Schizophrenia: A Meta-Analysis of Functional Imaging Studies. Acta Psychiatr. Scand..

[149] Glahn D.C., Ragland J.D., Abramoff A., Barrett J., Laird A.R., Bearden C.E., Velligan D.I. (2005). Beyond Hypofrontality: A Quantitative Meta-Analysis of Functional Neuroimaging Studies of Working Memory in Schizophrenia. Hum. Brain Mapp..

[150] Kühn S., Gallinat J. (2013). Resting-State Brain Activity in Schizophrenia and Major Depression: A Quantitative Meta-Analysis. Schizophr. Bull..

[151] Guimarães T.M., Machado-de-Sousa J.P., Crippa J.A.S., Guimarães M.R.C., Hallak J.E.C. (2016). Arterial Spin Labeling in Patients with Schizophrenia: A Systematic Review. Arch. Clin. Psychiatry.

[152] Laruelle M., Abi-Dargham A., Gil R., Kegeles L., Innis R. (1999). Increased Dopamine Transmission in Schizophrenia: Relationship to Illness Phases. Biol. Psychiatry.

[153] Abi-Dargham A., Rodenhiser J., Printz D., Zea-Ponce Y., Gil R., Kegeles L.S., Weiss R., Cooper T.B., Mann J.J., Van Heertum R.L. (2000). Increased Baseline Occupancy of D2 Receptors by Dopamine in Schizophrenia. Proc. Natl. Acad. Sci. USA.

[154] Poels E.M.P., Kegeles L.S., Kantrowitz J.T., Slifstein M., Javitt D.C., Lieberman J.A., Abi-Dargham A., Girgis R.R. (2014). Imaging Glutamate in Schizophrenia: Review of Findings and Implications for Drug Discovery. Mol. Psychiatry.

[155] Nikolaus S., Müller H.W., Hautzel H. (2016). Different Patterns of 5-HT Receptor and Transporter Dysfunction in Neuropsychiatric Disorders—A Comparative Analysis of in Vivo Imaging Findings. Rev. Neurosci..

[156] Egerton A., Modinos G., Ferrera D., McGuire P. (2017). Neuroimaging Studies of GABA in Schizophrenia: A Systematic Review with Meta-Analysis. Transl. Psychiatry.

[157] Cannon T.D., Chung Y., He G., Sun D., Jacobson A., van Erp T.G.M., McEwen S., Addington J., Bearden C.E., Cadenhead K. (2015). North American Prodrome Longitudinal Study Consortium. Progressive Reduction in Cortical Thickness as Psychosis Develops: A Multisite Longitudinal Neuroimaging Study of Youth at Elevated Clinical Risk. Biol. Psychiatry.

[158] Fusar-Poli P., Cappucciati M., Rutigliano G., Schultze-Lutter F., Bonoldi I., Borgwardt S., Riecher-Rössler A., Addington J., Perkins D., Woods S.W. (2015). At Risk or Not at Risk? A Meta-Analysis of the Prognostic Accuracy of Psychometric Interviews for Psychosis Prediction. World Psychiatry Off. J. World Psychiatr. Assoc. WPA.

[159] Koutsouleris N., Meisenzahl E.M., Davatzikos C., Bottlender R., Frodl T., Scheuerecker J., Schmitt G., Zetzsche T., Decker P., Reiser M. (2009). Use of Neuroanatomical Pattern Classification to Identify Subjects in At-Risk Mental States of Psychosis and Predict Disease Transition. Arch. Gen. Psychiatry.

[160] Mechelli A., Riecher-Rössler A., Meisenzahl E.M., Tognin S., Wood S.J., Borgwardt S.J., Koutsouleris N., Yung A.R., Stone J.M. (2011). Neuroanatomical Abnormalities That Predate the Onset of Psychosis: A Multicenter Study. Arch. Gen. Psychiatry.

[161] Koutsouleris N., Riecher-Rössler A., Meisenzahl E.M., Smieskova R., Studerus E., Kambeitz-Ilankovic L., von Saldern S., Cabral C., Reiser M., Falkai P. (2015). Detecting the Psychosis Prodrome across High-Risk Populations Using Neuroanatomical Biomarkers. Schizophr. Bull..

[162] Cao H., Chén O.Y., Chung Y., Forsyth J.K., McEwen S.C., Gee D.G., Bearden C.E., Addington J., Goodyear B., Cadenhead K.S. (2018). Cerebello-Thalamo-Cortical Hyperconnectivity as a State-Independent Functional Neural Signature for Psychosis Prediction and Characterization. Nat. Commun..

[163] Collin G., Seidman L.J., Keshavan M.S., Stone W.S., Qi Z., Zhang T., Tang Y., Li H., Anteraper S.A., Niznikiewicz M.A. (2020). Functional Connectome Organization Predicts Conversion to Psychosis in Clinical High-Risk Youth from the SHARP Program. Mol. Psychiatry.

[164] Demjaha A., Murray R.M., McGuire P.K., Kapur S., Howes O.D. (2012). Dopamine Synthesis Capacity in Patients with Treatment-Resistant Schizophrenia. Am. J. Psychiatry.

[165] Guimond S., Béland S., Lepage M. (2018). Strategy for Semantic Association Memory (SESAME) Training: Effects on Brain Functioning in Schizophrenia. Psychiatry Res. Neuroimag..

[166] Cui L.B., Cai M., Wang X.R., Zhu Y.Q., Wang L.X., Xi Y.B., Wang H.N., Zhu X., Yin H. (2019). Prediction of Early Response to Overall Treatment for Schizophrenia: A Functional Magnetic Resonance Imaging Study. Brain Behav..

[167] Jauhar S., Veronese M., Nour M.M., Rogdaki M., Hathway P., Turkheimer F.E., Stone J., Egerton A., McGuire P., Kapur S. (2019). Determinants of Treatment Response in First-Episode Psychosis: An 18F-DOPA PET Study. Mol. Psychiatry.

[168] Tarcijonas G., Sarpal D.K. (2019). Neuroimaging Markers of Antipsychotic Treatment Response in Schizophrenia: An Overview of Magnetic Resonance Imaging Studies. Neurobiol. Dis..

[169] Pence A.Y., Pries L.K., Ferrara M., Rutten B.P.F., van Os J., Guloksuz S. (2022). Gender Differences in the Association between Environment and Psychosis. Schizophr. Res..

[170] Woolway G.E., Smart S.E., Lynham A.J., Lloyd J.L., Owen M.J., Jones I.R., Walters J.T.R., Legge S.E. (2022). Schizophrenia Polygenic Risk and Experiences of Childhood Adversity: A Systematic Review and Meta-Analysis. Schizophr. Bull..

[171] Large M., Mullin K., Gupta P., Harris A., Nielssen O. (2014). Systematic Meta-Analysis of Outcomes Associated with Psychosis and Co-Morbid Substance Use. Aust. N. Z. J. Psychiatry.

[172] Schoeler T., Petros N., Di Forti M., Pingault J.B., Klamerus E., Foglia E., Small A., Murray R., Bhattacharyya S. (2016). Association Between Continued Cannabis Use and Risk of Relapse in First-Episode Psychosis: A Quasi-Experimental Investigation Within an Observational Study. JAMA Psychiatry.

[173] Di Forti M., Marconi A., Carra E., Fraietta S., Trotta A., Bonomo M., Bianconi F., Gardner-Sood P., O’Connor J., Russo M. (2015). Proportion of Patients in South London with First-Episode Psychosis Attributable to Use of High Potency Cannabis: A Case-Control Study. Lancet Psychiatry.

[174] Di Forti M., Quattrone D., Freeman T.P., Tripoli G., Gayer-Anderson C., Quigley H., Rodriguez V., Jongsma H.E., Ferraro L., La Cascia C. (2019). EU-GEI WP2 Group. The Contribution of Cannabis Use to Variation in the Incidence of Psychotic Disorder across Europe (EU-GEI): A Multicentre Case-Control Study. Lancet Psychiatry.

[175] Ringen P.A., Nesvåg R., Helle S., Lagerberg T.V., Lange E.H., Løberg E.M., Agartz I., Andreassen O.A., Melle I. (2016). Premorbid Cannabis Use Is Associated with More Symptoms and Poorer Functioning in Schizophrenia Spectrum Disorder. Psychol. Med..

[176] Seddon J.L., Birchwood M., Copello A., Everard L., Jones P.B., Fowler D., Amos T., Freemantle N., Sharma V., Marshall M. (2016). Cannabis Use Is Associated with Increased Psychotic Symptoms and Poorer Psychosocial Functioning in First-Episode Psychosis: A Report From the UK National EDEN Study. Schizophr. Bull..

[177] Quattrone D., Di Forti M., Gayer-Anderson C., Ferraro L., Jongsma H.E., Tripoli G., La Cascia C., La Barbera D., Tarricone I., Berardi D. (2019). Transdiagnostic Dimensions of Psychopathology at First Episode Psychosis: Findings from the Multinational EU-GEI Study. Psychol. Med..

[178] Guloksuz S., Rutten B.P.F., Pries L.K., Ten Have M., de Graaf R., van Dorsselaer S., Klingenberg B., van Os J., Ioannidis J.P.A., European Network of National Schizophrenia Networks Studying Gene-Environment Interactions Work Package 6 (EU-GEI WP6) Group (2018). The Complexities of Evaluating the Exposome in Psychiatry: A Data-Driven Illustration of Challenges and Some Propositions for Amendments. Schizophr. Bull..

[179] Guloksuz S., van Os J., Rutten B.P.F. (2018). The Exposome Paradigm and the Complexities of Environmental Research in Psychiatry. JAMA Psychiatry.

[180] Pries L.K., Erzin G., Rutten B.P.F., van Os J., Guloksuz S. (2021). Estimating Aggregate Environmental Risk Score in Psychiatry: The Exposome Score for Schizophrenia. Front. Psychiatry.

[181] Erzin G., Pries L.K., van Os J., Fusar-Poli L., Delespaul P., Kenis G., Luykx J.J., Lin B.D., Richards A.L., Akdede B. (2021). Examining the Association between Exposome Score for Schizophrenia and Functioning in Schizophrenia, Siblings, and Healthy Controls: Results from the EUGEI Study. Eur. Psychiatry J. Assoc. Eur. Psychiatr..

[182] Pries L.K., Guloksuz S., Ten Have M., de Graaf R., van Dorsselaer S., Gunther N., Rauschenberg C., Reininghaus U., Radhakrishnan R., Bak M. (2018). Evidence That Environmental and Familial Risks for Psychosis Additively Impact a Multidimensional Subthreshold Psychosis Syndrome. Schizophr. Bull..

[183] Barzilay R., Calkins M.E., Moore T.M., Wolf D.H., Satterthwaite T.D., Cobb Scott J., Jones J.D., Benton T.D., Gur R.C., Gur R.E. (2019). Association between Traumatic Stress Load, Psychopathology, and Cognition in the Philadelphia Neurodevelopmental Cohort. Psychol. Med..

[184] Gaebel W., Falkai P., Hasan A. (2020). The Revised German Evidence- and Consensus-Based Schizophrenia Guideline. World Psychiatry Off. J. World Psychiatr. Assoc. WPA.

[185] Hasan A., Falkai P., Wobrock T., Lieberman J., Glenthoj B., Gattaz W.F., Thibaut F., Möller H.J., WFSBP Task force on Treatment Guidelines for Schizophrenia (2013). World Federation of Societies of Biological Psychiatry (WFSBP) Guidelines for Biological Treatment of Schizophrenia, Part 2: Update 2012 on the Long-Term Treatment of Schizophrenia and Management of Antipsychotic-Induced Side Effects. World J. Biol. Psychiatry Off. J. World Fed. Soc. Biol. Psychiatry.

[186] Remington G., Addington D., Honer W., Ismail Z., Raedler T., Teehan M. (2017). Guidelines for the Pharmacotherapy of Schizophrenia in Adults. Can. J. Psychiatry Rev. Can. Psychiatr..

[187] Keepers G.A., Fochtmann L.J., Anzia J.M., Benjamin S., Lyness J.M., Mojtabai R., Servis M., Walaszek A., Buckley P., Lenzenweger M.F. (2020). The American Psychiatric Association Practice Guideline for the Treatment of Patients with Schizophrenia. Am. J. Psychiatry.

[188] National Institute for Health and Care Excellence: Guidelines (2014). Psychosis and Schizophrenia in Adults: Prevention and Management.

[189] Hasan A., Falkai P., Wobrock T., Lieberman J., Glenthoj B., Gattaz W.F., Thibaut F., Möller H.J., World Federation of Societies of Biological Psychiatry (WFSBP) Task Force on Treatment Guidelines for Schizophrenia (2012). World Federation of Societies of Biological Psychiatry (WFSBP) Guidelines for Biological Treatment of Schizophrenia, Part 1: Update 2012 on the Acute Treatment of Schizophrenia and the Management of Treatment Resistance. World J. Biol. Psychiatry Off. J. World Fed. Soc. Biol. Psychiatry.

[190] Verdoux H., Tournier M., Bégaud B. (2010). Antipsychotic Prescribing Trends: A Review of Pharmaco-Epidemiological Studies. Acta Psychiatr. Scand..

[191] Taipale H., Tanskanen A., Mehtälä J., Vattulainen P., Correll C.U., Tiihonen J. (2020). 20-Year Follow-up Study of Physical Morbidity and Mortality in Relationship to Antipsychotic Treatment in a Nationwide Cohort of 62,250 Patients with Schizophrenia (FIN20). World Psychiatry Off. J. World Psychiatr. Assoc. WPA.

[192] Ostuzzi G., Vita G., Bertolini F., Tedeschi F., De Luca B., Gastaldon C., Nosé M., Papola D., Purgato M., Del Giovane C. (2022). Continuing, Reducing, Switching, or Stopping Antipsychotics in Individuals with Schizophrenia-Spectrum Disorders Who Are Clinically Stable: A Systematic Review and Network Meta-Analysis. Lancet Psychiatry.

[193] Huhn M., Arndt T., Schneider-Thoma J., Leucht S. (2022). Effects of Antipsychotics on Heart Rate in Treatment of Schizophrenia: A Systematic Review and Meta-Analysis. Ther. Adv. Psychopharmacol..

[194] McCutcheon R.A., Krystal J.H., Howes O.D. (2020). Dopamine and Glutamate in Schizophrenia: Biology, Symptoms and Treatment. World Psychiatry.

[195] Llorca P.M., Nuss P., Fakra É., Alamome I., Drapier D., El Hage W., Jardri R., Mouchabac S., Rabbani M., Simon N. (2022). Place of the Partial Dopamine Receptor Agonist Aripiprazole in the Management of Schizophrenia in Adults: A Delphi Consensus Study. BMC Psychiatry.

[196] Osugo M., Whitehurst T., Shatalina E., Townsend L., O’Brien O., Mak T.L.A., McCutcheon R., Howes O. (2022). Dopamine Partial Agonists and Prodopaminergic Drugs for Schizophrenia: Systematic Review and Meta-Analysis of Randomized Controlled Trials. Neurosci. Biobehav. Rev..

[197] Kumar V., Manchegowda S., Jacob A., Rao N.P. (2020). Glutamate Metabolites in Treatment Resistant Schizophrenia: A Meta-Analysis and Systematic Review of 1H-MRS Studies. Psychiatry Res. Neuroimag..

[198] Kiemes A., Davies C., Kempton M.J., Lukow P.B., Bennallick C., Stone J.M., Modinos G. (2021). GABA, Glutamate and Neural Activity: A Systematic Review with Meta-Analysis of Multimodal 1H-MRS-FMRI Studies. Front. Psychiatry.

[199] Bighelli I., Salanti G., Huhn M., Schneider-Thoma J., Krause M., Reitmeir C., Wallis S., Schwermann F., Pitschel-Walz G., Barbui C. (2018). Psychological Interventions to Reduce Positive Symptoms in Schizophrenia: Systematic Review and Network Meta-Analysis. World Psychiatry Off. J. World Psychiatr. Assoc. WPA.

[200] Barnicot K., Michael C., Trione E., Lang S., Saunders T., Sharp M., Crawford M.J. (2020). Psychological Interventions for Acute Psychiatric Inpatients with Schizophrenia-Spectrum Disorders: A Systematic Review and Meta-Analysis. Clin. Psychol. Rev..

[201] Todorovic A., Lal S., Dark F., De Monte V., Kisely S., Siskind D. (2020). CBTp for People with Treatment Refractory Schizophrenia on Clozapine: A Systematic Review and Meta-Analysis. J. Ment. Health Abingdon Engl..

[202] Mc Glanaghy E., Turner D., Davis G.A., Sharpe H., Dougall N., Morris P., Prentice W., Hutton P.A. (2021). Network Meta-Analysis of Psychological Interventions for Schizophrenia and Psychosis: Impact on Symptoms. Schizophr. Res..

[203] Gastaldon C., Mosler F., Toner S., Tedeschi F., Bird V.J., Barbui C., Priebe S. (2019). Are Trials of Psychological and Psychosocial Interventions for Schizophrenia and Psychosis Included in the NICE Guidelines Pragmatic? A Systematic Review. PLoS ONE.

[204] Nucifora F.C., Woznica E., Lee B.J., Cascella N., Sawa A. (2019). Treatment Resistant Schizophrenia: Clinical, Biological, and Therapeutic Perspectives. Neurobiol. Dis..

[205] Corripio I., Roldán A., Sarró S., McKenna P.J., Alonso-Solís A., Rabella M., Díaz A., Puigdemont D., Pérez-Solà V., Álvarez E. (2020). Deep Brain Stimulation in Treatment Resistant Schizophrenia: A Pilot Randomized Cross-over Clinical Trial. EBioMedicine.

[206] Sciortino D., Pigoni A., Delvecchio G., Maggioni E., Schiena G., Brambilla P. (2021). Role of RTMS in the Treatment of Cognitive Impairments in Bipolar Disorder and Schizophrenia: A Review of Randomized Controlled Trials. J. Affect. Disord..

[207] Glenthøj L.B., Mariegaard L.S., Fagerlund B., Jepsen J.R.M., Kristensen T.D., Wenneberg C., Krakauer K., Medalia A., Roberts D.L., Hjorthøj C. (2020). Effectiveness of Cognitive Remediation in the Ultra-High Risk State for Psychosis. World Psychiatry Off. J. World Psychiatr. Assoc. WPA.

[208] Lysaker P.H., Hasson-Ohayon I. (2021). Metacognition in Psychosis: A Renewed Path to Understanding of Core Disturbances and Recovery-oriented Treatment. World Psychiatry.

[209] Phelan S., Sigala N. (2022). The Effect of Treatment on Insight in Psychotic Disorders—A Systematic Review and Meta-Analysis. Schizophr. Res..

[210] Schroeder A.H., Bogie B.J.M., Rahman T.T., Thérond A., Matheson H., Guimond S. (2022). Feasibility and Efficacy of Virtual Reality Interventions to Improve Psychosocial Functioning in Psychosis: Systematic Review. JMIR Ment. Health.

[211] Rodolico A., Bighelli I., Avanzato C., Concerto C., Cutrufelli P., Mineo L., Schneider-Thoma J., Siafis S., Signorelli M.S., Wu H. (2022). Family Interventions for Relapse Prevention in Schizophrenia: A Systematic Review and Network Meta-Analysis. Lancet Psychiatry.

[212] Bond G.R., Drake R.E., Becker D.R. (2020). An Update on Individual Placement and Support. World Psychiatry.

[213] Hellström L., Pedersen P., Christensen T.N., Wallstroem I.G., Bojesen A.B., Stenager E., Bejerholm U., van Busschbach J., Michon H., Mueser K.T. (2021). Vocational Outcomes of the Individual Placement and Support Model in Subgroups of Diagnoses, Substance Abuse, and Forensic Conditions: A Systematic Review and Analysis of Pooled Original Data. J. Occup. Rehabil..

[214] Frederick D.E., VanderWeele T.J. (2019). Supported Employment: Meta-Analysis and Review of Randomized Controlled Trials of Individual Placement and Support. PLoS ONE.

[215] Brinchmann B., Widding-Havneraas T., Modini M., Rinaldi M., Moe C.F., McDaid D., Park A.L., Killackey E., Harvey S.B., Mykletun A.A. (2020). Meta-Regression of the Impact of Policy on the Efficacy of Individual Placement and Support. Acta Psychiatry Scand..

[216] Baller J.B., Blyler C.R., Bronnikov S., Xie H., Bond G.R., Filion K., Hale T. (2020). Long-Term Follow-Up of a Randomized Trial of Supported Employment for SSDI Beneficiaries with Mental Illness. Psychiatry Serv..

[217] Feldman R. (2020). What Is Resilience: An Affiliative Neuroscience Approach. World Psychiatry.

[218] Harvey P.D., Strassing M. (2012). Predicting the Severity of Everyday Functional Disability in People with Schizophrenia: Cognitive Deficits, Functional Capacity, Symptoms, and Health Status. World Psychiatry.

[219] Green M.F., Horan W.P., Lee J. (2019). Nonsocial and Social Cognition in Schizophrenia: Current Evidence and Future Directions. World Psychiatry Off. J. World Psychiatr. Assoc. WPA.

[220] Galderisi S., Rucci P., Mucci A., Rossi A., Rocca P., Bertolino A., Aguglia E., Amore M., Bellomo A., Bozzatello P. (2020). Italian Network for Research on Psychoses. The Interplay among Psychopathology, Personal Resources, Context-Related Factors and Real-Life Functioning in Schizophrenia: Stability in Relationships after 4 Years and Differences in Network Structure between Recovered and Non-Recovered Patients. World Psychiatry Off. J. World Psychiatr. Assoc. WPA.

[221] Jablensky A. (2016). Psychiatric Classifications: Validity and Utility. World Psychiatry.

[222] Kendler K.S. (2016). The Nature of Psychiatric Disorders. World Psychiatry Off. J. World Psychiatr. Assoc. WPA.

[223] Comparelli A., Raballo A., Pompili M., Galderisi S. (2019). Beyond the Transnosographic Emphasis on Psychosis: Nosological Perspectives on Schizophrenia and Its Prevention. Front. Psychiatry.

[224] Martínez-Alés G., Susser E.S. (2022). A Useful Construct to Improve the Lives of People with Schizophrenia. Schizophr. Res..

[225] Maruta T., Volpe U., Gaebel W., Matsumoto C., Iimori M. (2014). Should Schizophrenia Still Be Named So?. Schizophr. Res..

[226] Nasrallah H.A. (2021). To Change the Label of Schizophrenia, First Revise the Construct. Schizophr. Res..

[227] Yamaguchi S., Mizuno M., Ojio Y., Sawada U., Matsunaga A., Ando S., Koike S. (2017). Associations between Renaming Schizophrenia and Stigma-Related Outcomes: A Systematic Review. Psychiatry Clin. Neurosci..

[228] Mesholam-Gately R.I., Varca N., Spitzer C., Parrish E.M., Hogan V., Behnke S.H., Larson L., Rosa-Baez C., Schwirian N., Stromeyer C. (2021). Are We Ready for a Name Change for Schizophrenia? A Survey of Multiple Stakeholders. Schizophr. Res..

[229] Chiu H.F.K., Sato M., Kua E.H., Lee M.S., Yu X., Ouyang W.C., Yang Y.K., Sartorius N. (2014). Renaming Dementia—An East Asian Perspective. Int. Psychogeriatr..

[230] Sato M. (2006). Renaming Schizophrenia: A Japanese Perspective. World Psychiatry Off. J. World Psychiatr. Assoc. WPA.

[231] Cho J.W., Jang E.Y., Woo H.J., Park Y.C., Kim S.H., Hong K.S., Lee Y.S., Kwon J.S. (2018). Effects of Renaming Schizophrenia in Korea: From “Split-Mind Disorder” to “Attunement Disorder”. Psychiatry Investig..

[232] Koike S., Yamaguchi S., Ohta K., Ojio Y., Watanabe K.I., Ando S. (2017). Mental-Health-Related Stigma among Japanese Children and Their Parents and Impact of Renaming of Schizophrenia. Psychiatry Clin. Neurosci..

[233] Lieberman J.A., First M.B. (2007). Renaming Schizophrenia. BMJ.

[234] Lasalvia A., Penta E., Sartorius N., Henderson S. (2015). Should the Label “Schizophrenia” Be Abandoned?. Schizophr. Res..

[235] Gaebel W., Kerst A. (2018). The Debate about Renaming Schizophrenia: A New Name Would Not Resolve the Stigma. Epidemiol. Psychiatry Sci..

